# Progress in topical nanoformulations against bacterial skin and soft tissue infections– current trends

**DOI:** 10.1007/s13346-025-01924-7

**Published:** 2025-07-19

**Authors:** Michelle Zhi Yee Teo, Hooi Leong Loo, Bey Hing Goh, Lay Hong Chuah

**Affiliations:** 1 School of Pharmacy, Monash University Malaysia, Bandar Sunway, Subang Jaya, Selangor 47500 Malaysia; 2 Sunway Biofunctional Molecules Discovery Centre (SBMDC), Faculty of Medical and Life Sciences, Sunway University, Sunway City, Selangor Malaysia; 3 Australian Research Centre in Complementary and Integrative Medicine, Faculty of Health, University of Technology Sydney, Ultimo, NSW Australia; 4 Biofunctional Molecule Exploratory Research Group, School of Pharmacy, Monash University Malaysia, Selangor Darul Ehsan, Bandar Sunway, Subang Jaya, 47500 Sunway Malaysia; 5 Graduate Institute of Cancer Biology and Drug Discovery, College of Medical Science and Technology, Taipei Medical University, Taipei, 11031 Taiwan

**Keywords:** Nanoformulations, Nanomedicine, Skin infection, Bacterial infections, Topical, Dermal, Nanoparticles, Nanoemulsions, Nanovesicles

## Abstract

The accelerating rate of antibiotic resistance has always been one of the leading causes of increased skin and soft tissue infections (SSTIs) burden around the globe. Current treatments mainly focus on systemic antibiotics indicated for both uncomplicated and complicated SSTIs that act as a contributing factor secondary to widespread systemic exposure. Topical formulation of antibacterial agents or antibiotics are renowned for their targeted and localised action in the skin which appears as an intriguing clue to the resistance problem. Nevertheless, there are several deterrents associated with conventional topical formulations including drug permeability and skin retention. This has propelled the transformation of SSTI intervention towards the incorporation of nanotechnology to enhance topical drug delivery for SSTIs. This review outlines the advancement of nanoparticle-based topical formulations against SSTIs, covering cellulitis and erysipelas, boils and carbuncles, impetigo, cutaneous non-tuberculous mycobacterial infections and leprosy, as well as pitted keratolysis. Pre-clinical safety profile and antibacterial efficacy of topical nanoformulations were comprehensively reviewed and classified into multiple categories such as metal nanoparticles, emulsion-based nanosystems, nanovesicles, lipid nanoparticles and polymeric nanoparticles. The up-to-date patent trends on topical nanoformulations for SSTIs up to 2025 were also analysed and justified based on current evidence to pinpoint the research gap and future prospects in this growing area of research. It is anticipated that topical nanoformulations can potentially stand in for conventional topical formulations to treat SSTIs attributed to their pronounced antibacterial activity and tolerability.

## Graphical Abstract

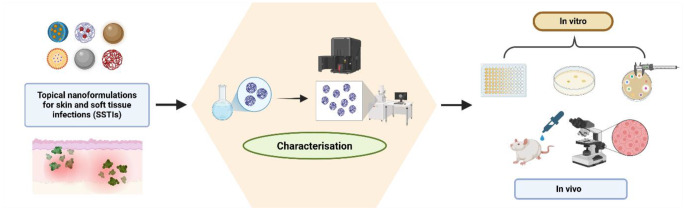

## Introduction

**Fig. 1:**
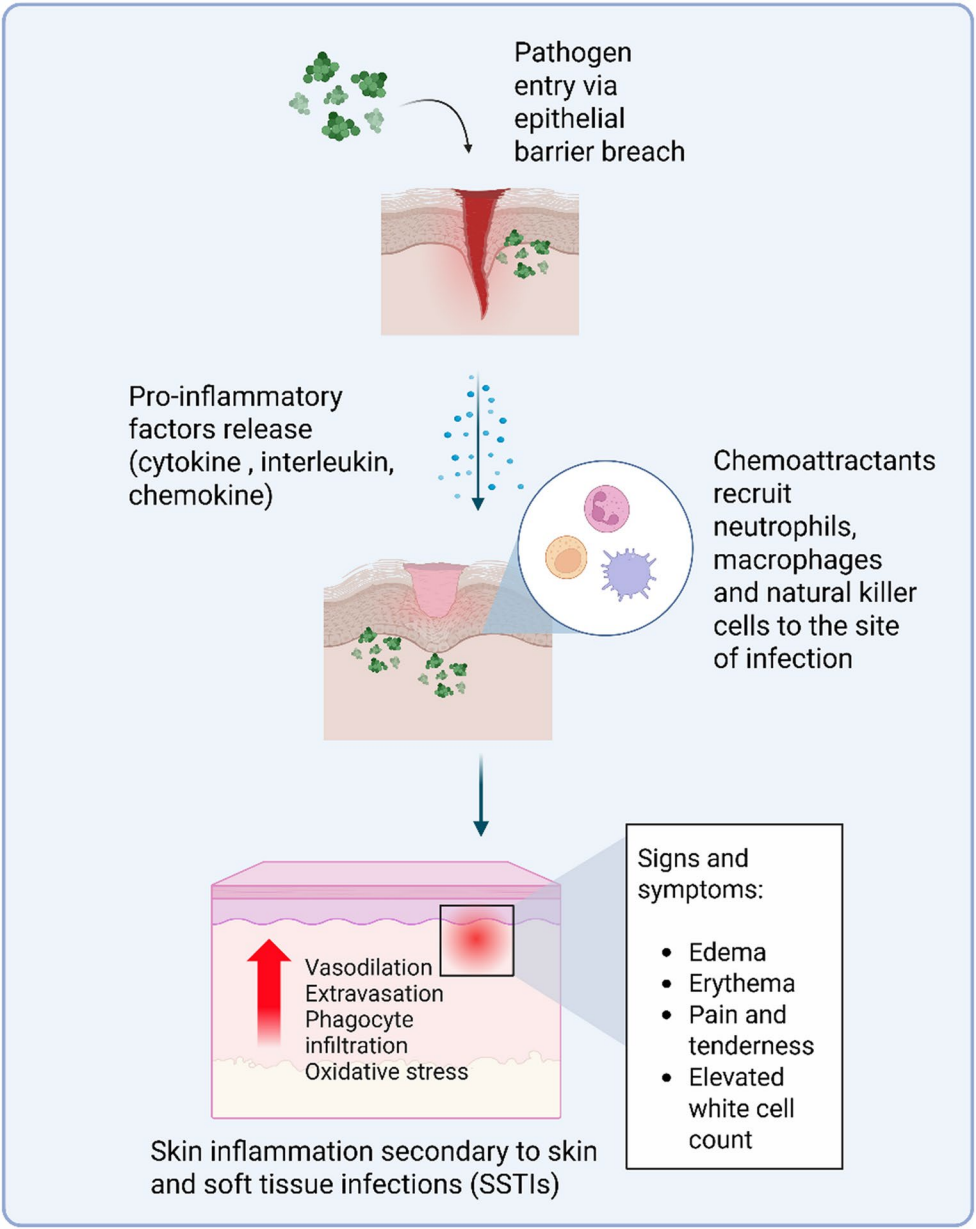
General pathophysiology of skin and soft tissue infections (SSTIs)

Skin and soft tissue infections (SSTIs) encompass a group of infections that involve one of the major organs of our body, the skin, where the affliction is usually caused by bacterial pathogens [1]. Common clinical presentations of SSTIs comprise of impetigo, leprosy, pitted keratolysis, cellulitis, erysipelas, boils, and carbuncles to name a few [2]. These clinical presentations can be further classified as uncomplicated or complicated depending on the nature of the disease and disease prognosis [1, 2]. Typically, the general pathophysiology of SSTIs starts with the invasion of a pathogen into the host system via any entry point of the skin as illustrated in Fig. 1. Some of the common causative pathogens are unsurprisingly, normal flora residing on the skin [3]. When the balance of the skin microbiome is being disrupted secondary to extrinsic factors (e.g. colonisation of new bacteria, trauma, environment, etc.) or intrinsic factors (e.g. being immunocompromised, hormonal imbalances, etc.), there will be augmented risk of SSTI acquisition [4, 5]. Examples of normal flora include *Staphylococcus* spp. and *Micrococcus* spp. that are found abundantly on the skin which may turn pathogenic and result in SSTIs such as cellulitis and pitted keratolysis [3].

In 2019, Global Burden of Disease Study (GBD) reported the number of bacterial skin disease to be around 146.84 million cases globally [6]. The annualised bacterial SSTIs incidence rate increased by 7.38% from 1990 to 2019 based on the estimation using GBD 2019 [6]. Notably, the heaviest region-specific burden was 294.74 million incidences in Southern Sub-Saharan Africa, while high-income North America had the lowest burden with 46.37 million cases in 2019 [6]. It is believed that the trend of SSTI disease burden is constantly increasing particularly with multi-drug-resistant bacteria becoming more and more ubiquitous over years [7, 8].

Most of the treatment options recommended by current guidelines for SSTIs revolve around systemic antibiotics given either orally or intravenously. While topical antibiotic formulations are seldom used, and when used would typically be in conjunction with a systemic antibiotic [9]. This common practice is likely associated with concern surrounding suboptimal drug concentration below the therapeutic threshold, especially with compromised skin integrity which loses its ability for effective permeation and drug deposition [10]. Another possible reason is the physicochemical properties of antibiotics that ultimately influences the absorption and distribution across the skin, which in turn greatly impacts the treatment outcome [10]. On the other hand, several downsides remain with existing antibiotic regimens, namely the emergence of antibiotic resistance and increased risk of antibiotic-related systemic toxicity [11, 12]. Introduction of antibiotics into the blood circulation when there are no associated SSTIs systemic features may be more likely to cause antibiotic resistance without improving treatment effectiveness. Aside from the remarkable antibacterial activity in eradicating the causative pathogens, systemic treatments can also disrupt the microbiota within the host, potentially leading to the development of secondary infections, also known as superinfections (e.g. infective endocarditis, osteomyelitis, necrotising fasciitis, etc.) [12].

In recent years, nanotechnology has been in high-demand and extensively assimilated in a myriad of fields including engineering, biomedicine, cosmetics, textiles, material sciences, not to mention pharmaceutical sciences [13]. In the context of topical dermal applications, the most prominent practice is in wound management aiming to develop wound healing materials with antimicrobial properties [14]. In addition to ongoing efforts in antimicrobial stewardship and new drug development, research shows that using antimicrobial agents topically can help improve current treatments for skin infections and lower the risk of antimicrobial resistance [15, 16]. To achieve this, nanotechnology can be turned to as a promising platform with a substantial volume of research surrounding its biomedical use [17, 18].

This review aims to critically evaluate the available literature surrounding the pre-clinical development of nanoparticle-based topical drug formulations against bacterial SSTIs while highlighting the connections between the academic research and translational aspects in terms of patent trends and clinical evidence. By scrutinising the pros and cons of topical nanoformulations in the treatment of SSTIs, this review offers new insights and suggests avenues for future research.

## Skin and soft tissue infections

### Cellulitis and erysipelas

**Fig. 2:**
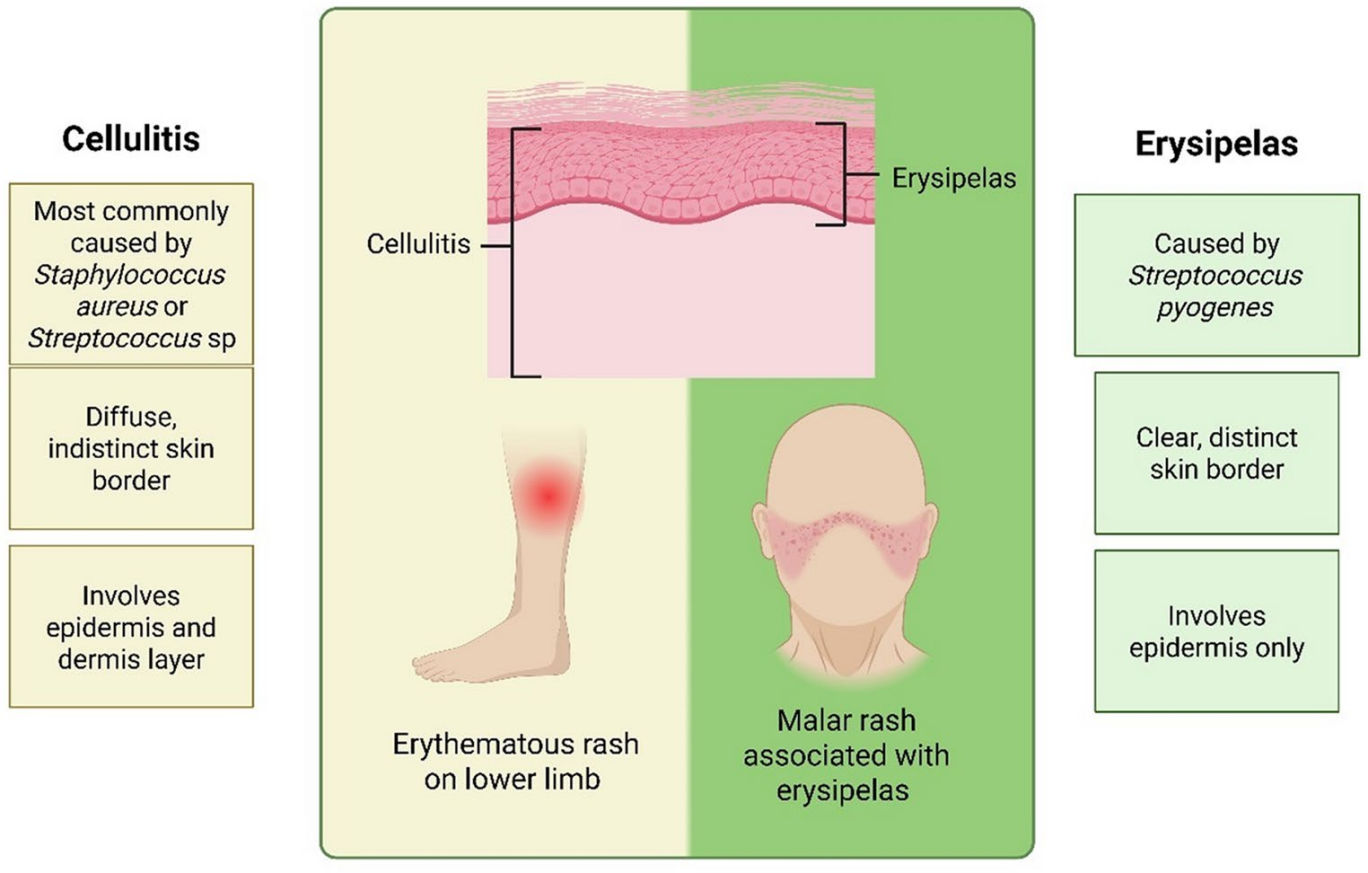
Differential diagnosis of cellulitis and erysipelas

Cellulitis is a deep dermal infection involving the epidermis, dermis and subcutaneous layer with various presentations, mostly caused by Gram-positive bacteria such as *Staphylococcus* spp. and *Streptococcus* spp [4, 5, 19]. In the US, there are more than 14 million cases of cellulitis that contributes to 650,000 hospitalisations every year and is responsible for 1 to 2% of emergency department visits [20, 21]. *Staphylococcus aureus* (*S. aureus*) is more likely to be found in purulent cellulitis with the presence of abscess, ulceration, penetrating trauma, and purulent discharge [4]. Non-purulent, spontaneous or rapidly spreading cellulitis is triggered by *Streptococcus pyogenes* (*S. pyogenes*), or another *Streptococcus* spp. (i.e. group B, C or G) [4, 5]. Rarely, Gram-negative bacteria such as *Pseudomonas aeruginosa* (*P. aeruginosa*) are found to be the causative pathogen, however, immunocompromised individuals may be at a higher risk, as they would typically be at the threat of incurring an infection from a broader range of microbes than immunocompetent individuals [3–5].

Erysipelas is a superficial dermal infection predominantly caused by *S. pyogenes* (group A *streptococcus*) that only affects the epidermis [3, 4, 19]. Erysipelas is more prevalent amongst infants, young children and elderly [22]. Both cellulitis and erysipelas share common features such as symptoms manifesting around the lower limbs and are usually unilateral, associated with warmness, erythema, tenderness, and swelling (Fig. 2) [3–5]. Systemic features including fever, lymphadenopathy, tachycardia, and shortness of breath can also present in severe cases [2]. Cellulitis presents with slightly raised skin with diffuse or indistinct borders while erysipelas has clear, distinct borders surrounding the marked raised skin due to inflammation that can be distinguished from cellulitis [19]. Additionally, erysipelas often presents with a unique butterfly rash on face appearance [3, 23].

According to the recommendation issued by the Centers for Disease Control and Prevention (CDC), systemic antibiotics are known as the first-line intervention for both cellulitis and erysipelas [24]. Oral antibiotics can be given as empirical therapy for patients without systemic features including fever, tachypnoea, tachycardia, and abnormal white cell counts. If there are two or more systemic symptoms, intravenous antibiotic should be administered [5, 19, 24].

### Boils and carbuncles

Boils and carbuncles are bacterial infections that occur within the vicinity of hair follicles, which are usually noticed on the axillae, groin, and buttocks in clusters [3]. Boils are also called furuncles with clinical presentation of a single head, while carbuncles are characterized by multiple heads [3]. Both are associated with formation of pus-discharging cutaneous abscess extending to the subcutaneous layer, tenderness, and pain [3, 4]. There were at least 280,000 boil episodes reported worldwide in 2010 [25]. The most common causative pathogen is Gram-positive *S. aureus*, sometimes occurring in combination with *S. pyogenes* to a lesser extent [3, 4].

Oral antibiotic therapy is usually only considered in conjunction with incision and drainage for larger abscesses (more than 5 cm in diameter), diabetic patients, recurrent infection, and systemic inflammatory response syndrome (SIRS) [26, 27]. A meta-analysis of over 589 patients in 2013 concluded that adjunctive systemic antibiotics in addition to incision and drainage do not significantly improve the rate of complete resolution of simple abscesses (*P* > 0.05), which was defined as having no subsequent surgical procedures required [28]. On the other hand, a randomised controlled trial conducted in the US found that adjunctive antibiotics provided significant benefits in treating skin abscesses. Specifically, the use of trimethoprim-sulfamethoxazole led to a higher clinical and composite cure rate compared to the placebo group. These benefits were observed regardless of whether the patients met the guideline-recommended indications for antibiotic use [27]. Routine systemic antibiotics are typically unnecessary in the absence of systemic features.

### Impetigo

Impetigo is a superficial bacterial SSTI known to be highly contagious, commonly occurring in children more than adults [5]. At a certain point, impetigo affected roughly up to 162 million children globally as estimated in a study by Bowen et al., predominantly in the Indigenous Australian population [29]. Other than young age, environmental factors including poor housing area, crowded conditions, warm and humid weather poses a higher risk of acquiring impetigo [3, 5]. Untreated impetigo can potentially result in serious complications such as acute rheumatic fever, rheumatic heart disease, and sepsis, contributing to a mortality rate of 5 to 10% [30]. Impetigo is caused by Gram-positive bacteria that can be clinically presented as non-bullous (crusted) or bullous [3, 5]. Bullous impetigo involves rapid erosion of blisters and is mainly caused by *S. aureus* whereas non-bullous impetigo usually has a yellow crust and erosion without pain but itchiness [5]. Epidemiology and setting of acquisition will determine the suspected causative pathogen, as such, *S. aureus* is more common in non-endemic settings while *S. pyogenes* prevails in endemic settings (remote communities) [29, 30].

Topical antibiotics are recommended to treat impetigo with localised skin sores in a non-endemic setting [31]. For instance, topical 2% fusidic acid (FA) or 2% mupirocin which has therapeutic effectiveness equivalent or superior to oral antibiotic as proposed by Koning et al. [31]. Nevertheless, prescribing systemic antibiotics is a common practice for impetigo in endemic and non-endemic settings when there is presence of multiple skin sores [26, 29–31]. For generalised impetigo, also known as ecthyma, systemic antibiotics are preferred [31].

### Cutaneous non-tuberculous mycobacterial infections and leprosy

The *Mycobacterium* genus encompasses the *Mycobacterium tuberculosis* (MTB) complex, *Mycobacterium leprae*, and non-tuberculous mycobacteria (NTM) [32]. NTMs are opportunistic pathogens known to cause localised SSTIs [33, 34]. These pathogens are classified based on colony formation time, identified as either rapid-growing (< 7 days) or slow-growing (≥ 7 days) species [33]. Rapid-growing mycobacteria (RGM) implicated in SSTIs include *Mycobacterium fortuitum*, *Mycobacterium abscessus*, and *Mycobacterium chelonae*, while slow-growing species include *Mycobacterium marinum* (associated with fish tank granuloma), *Mycobacterium ulcerans* (causing Buruli ulcers), and *Mycobacterium haemophilum* [33]. NTM-related SSTIs are frequently associated with nosocomial infections following surgery, trauma, or cosmetic procedures (e.g., liposuction, mesotherapy, tattooing), due to their capacity for biofilm formation, which contributes to their high resistance profile [35].

The global epidemiology of NTM SSTIs remains unclear, with available data largely limited to regional studies. This limitation is primarily due to the complex clinical presentation and prolonged incubation phase of the disease, which complicate definitive diagnosis [36]. Between 1996 and 2017, NTM SSTIs accounted for 15.3% of 365 NTM cases identified in Iowa, United States [37]. In China, a recent retrospective study at a tertiary hospital reported a statistically significant annual increase in NTM infections from 2014 to 2023 (*P* < 0.01) [36]. The clinical manifestations of NTM SSTIs such as abscesses, sporotrichoid nodules, or ulcers are not pathognomonic and often overlap with other infectious dermatoses, including sporotrichosis, cutaneous tuberculosis, nocardiosis, cutaneous leishmaniasis, aspergillosis, cryptococcosis, and histoplasmosis [34, 36, 38]. Treatment typically involves a combination of a systemic macrolide with a fluoroquinolone, doxycycline, or trimethoprim-sulfamethoxazole, in accordance with the American Thoracic Society and the Infectious Diseases Society of America (ATS/IDSA) guidelines [39].

Leprosy or Hansen’s disease is a neglected tropical disease caused by *Mycobacterium leprae* which can affect the skin, eye, respiratory tract, bone, liver and peripheral nervous system [40]. Leprosy is known to be prevalent across at least 122 countries and there were over 5 million cases around the globe in the 1980s [41]. The World Health Organization (WHO) declared the successful eradication of leprosy as a global health issue in the year 2000 based on the World Health Assembly resolution 44.9 by achieving a prevalence of less than 1 per 10 000 population [42]. Between 2005 and 2014, the global incidence rate declined to about 200,000 [43]. Pronounced decline in worldwide prevalence was observed with less than 129,192 leprosy cases during late 2020s after the implementation of WHO disease control. These efforts include contact tracing, post-exposure prophylaxis with a single dose of rifampicin, case detection and multi-drug therapy, health awareness and education [41, 42].

The disease is characterised by skin discolouration, nodules, patches, and painless ulcers or lumps which are hypopigmented [41–43]. Some infected individuals may experience numbness around the area of the affected skin, muscle weakness, paralysis in hands and feet, or enlarged nerves visualised at elbows, knees and neck, indicating nerve damage caused by the infection [40]. Damage in ocular nerves can also result in blindness [40]. Treatment regimens and clinical prognosis are guided by the leprosy classification system established by the WHO in 1982 built from the Ridley-Jopling system since 1960 based on histopathological appearance [42]. Leprosy is a communicable disease but also curable, especially upon early initiation of treatment [40, 42]. WHO divided leprosy manifestations dependent on cell-mediated immune response into three categories from the least severe to most severe, tuberculoid (strong), borderline (variable), lepromatous (deficient) [42]. Combination of oral rifampicin, dapsone, and clofazimine constitutes the backbone of leprosy treatment [43].

### Pitted keratolysis

Pitted keratolysis is a superficial bacterial skin infection that is caused by Gram-positive bacteria such as *Actinomyces* sp., *Corynebacteria* sp., *Dermatophilus congolensis*,* Kytococcus sedentarious* (formerly known as *Micrococcus sedentarious*) and *Streptomyces* sp. that affects the plantar surface, commonly the pressure-bearing areas [44–46]. Hot and humid environments promote proliferation and outgrowth of those bacteria and thus poses as occupational risk factors for individuals who are exposed to prolonged footwear occlusion including farmers, paddy field workers, soldiers, athletes, and marine workers [47]. The pathophysiology involves the action of bacterial protease enzymes, keratin-degrading serine proteases, which destroy the structural skin layer of the stratum corneum via keratin degradation. This leads to consequential characteristic features such as the formation of crater-like pits (small indentations) on the soles, heels, between toes with or without pruritus, pain and hyperhidrosis [45, 47]. Infected feet can be malodorous due to production of sulphur compounds such as thiols, sulphides and thioesters by the causative pathogens. In more serious cases, numerous vivid pits coalesce into lesions, presenting as irregular erosions or sulci in a diverse range of diameters (0.5 to 7.0 mm) and depths (1 to 2 mm) with or without erythema [45–47].

The disease is treatable, non-contagious, and non-inflammatory unlike other SSTIs discussed above, thus has an excellent prognosis where the indentations and odour can be cured within 1 month by effective antibiotic treatment [46]. Topical antibiotics are the recommended first-line treatments for pitted keratolysis including erythromycin solution or gel, FA cream, mupirocin cream and clindamycin solution [47]. Oral antibiotics are only indicated for those cases refractory to topical antibiotics [47].

## Constraints in antimicrobial management of skin and soft tissue infections

### Limitations in the current treatment modalities

Overuse and misuse of systemic antibiotics remain among the most significant deterrents in the current treatment modalities for SSTIs [10]. It is not peculiar to encounter hospital cases with prolonged antibiotic courses that exceed the guideline recommended duration for specific indications [48–50]. Even with limited supporting evidence, surgical antibiotic prophylaxis is often continued postoperatively to prevent surgical site infections [51]. The unnecessary use of antimicrobials elevates the risk of developing superinfections, which may occur either successively or as co-infections alongside the primary infection, caused by different pathogenic organisms [52]. These superinfections often involve pathogens resistant to the antimicrobial therapy initially administered for the primary infection [52]. Such overprescribing practices contribute to the emergence and dissemination of resistant bacterial strains, thereby complicating the effective management of SSTIs [51].

The emergence and spread of resistant strains compromise local susceptibility profiles and antibiograms, often prompting revisions to antimicrobial prescribing guidelines [9, 53, 54]. In regions with a high prevalence of antimicrobial resistance, broad-spectrum antibiotics are frequently employed as empirical therapy that could be potentially effective in the short term. This represents a temporary solution rather than a sustainable measure for disease control [11, 50, 55]. Examples of plausible ongoing efforts are drug repositioning and repurposing strategies, development of novel antimicrobial agents and the implementation of antimicrobial stewardship programs [55–57]. Meanwhile, an adaptation to the cardinal change in antimicrobial prescribing pattern is crucial to truncate the expansion of antimicrobial resistance, particularly to regions that have not yet been significantly affected [55–57].

It is essential for healthcare practitioners to critically assess and rationalise the need for systemic antibiotics in the treatment of uncomplicated SSTIs. Although topical antibiotics are recommended as first-line therapy for several SSTI indications, there remains a notable prescribing bias favouring oral antibiotics [12, 49]. This trend is often driven by public misconceptions regarding their superior effectiveness which may not be clinically justified in many cases [10, 12]. The direct escalation to systemic antibiotics in the absence of red flag symptoms can hasten the growing trend of antimicrobial resistance by increasing selection pressure across the host’s microbiota. Encouraging the appropriate use of topical antibiotics for uncomplicated SSTIs could play a pivotal role in curbing the rapid development of resistant strains [10, 57]. This approach upholds the antimicrobial stewardship goals and secures the systemic antibiotics as a last-resort option for managing severe or resistant infections.

### Barriers to conventional topical antimicrobial skin and soft tissue infection therapy

In addition to their suboptimal therapeutic outcomes, conventional topical dermal antimicrobial therapies for SSTIs are associated with several critical limitations. A major challenge is inadequate skin penetration, which restricts the delivery of antimicrobial agents to deeper skin layers such as the dermis and hypodermis, often implicated in more severe or deep-seated infections [58]. Furthermore, poor retention of topically applied drugs necessitates frequent reapplication, increasing the treatment burden and potentially reducing patient adherence which then contributes to treatment failure [58]. The use of certain active agents or formulation excipients may also elicit dermal irritation or hypersensitivity reactions, further compromising tolerability and compliance [59]. Collectively, these limitations may contribute to therapeutic failure and the emergence of antimicrobial resistance, underscoring the urgent need for improved topical delivery strategies to enhance the management of SSTIs and mitigate future public health risks.

Traditional innovation in antimicrobial therapy often focuses on the discovery of novel agents, which remains essential but is inherently time-consuming and costly, particularly in the context of rapidly evolving antimicrobial resistance [60]. However, the long-term efficacy of new antimicrobials may be compromised if the inherent limitations of topical delivery are not adequately addressed, thereby perpetuating a vicious cycle of treatment failure and resistance development. In contrast, the development of novel drug delivery systems represents a more sustainable, timely, and cost-effective strategy [61].

### Dermal delivery of topical antimicrobial for SSTIs

**Fig. 3:**
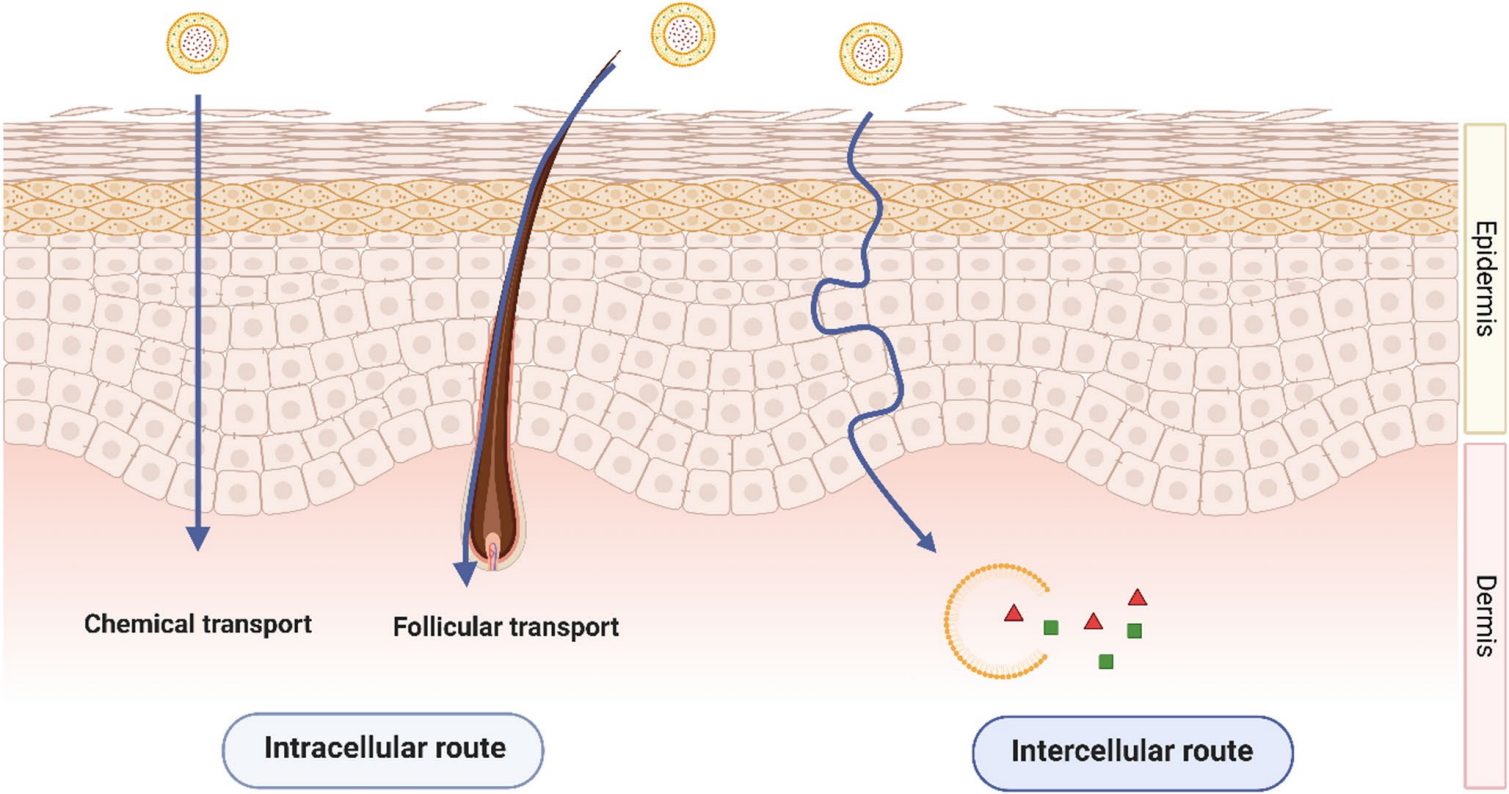
Mechanisms of skin penetration via dermal delivery of topical antimicrobials

Skin permeation and drug retention are key considerations in the development of topical nanoformulations for the treatment of uncomplicated SSTIs. The understood mechanisms of skin penetration are generally categorised into intercellular (via interstitial spaces) and intracellular routes (via biochemical interactions with the transport proteins and membrane lipids) including the skin appendages such as hair follicles, sweat glands, sebaceous glands, etc [62]. Among the critical nanoparticle characterisation parameters influencing topical drug permeability across dermal layers, particle size, lipophilicity, and the ionisation ratio, adapted from Lipinski’s Rule of Five, a traditional “rule of thumb” used to predict oral drug bioavailability based on physicochemical properties can be employed in a similar fashion [63]. Skin permeability is enhanced when nanoformulations increase membrane fluidity through molecular interactions with surface groups, enabling the active drug to reach the target site of uncomplicated SSTIs as illustrated in Fig. 3. Since the skin surface carries a net negative charge, nanoformulations with negative zeta potential can promote drug deposition by acting as a reservoir within the dermal layers while also facilitating controlled drug release [64]. Conversely, positively charged formulations may enhance skin retention via electrostatic interactions with the negatively charged skin surface [65].

### Considerations in developing topical antimicrobial nanoformulations for SSTIs

While the numerous advantages of topical dermal drug delivery are well-recognised, several challenges persist. Issues such as ineffective skin penetration, poor skin retention, and suboptimal drug deposition continue to deter the clinical application of topical antimicrobials [66]. Nanotechnology provides a promising solution by enabling the encapsulation of active drugs within specialised nanocarriers designed to enhance dermal delivery against uncomplicated SSTIs [67]. The small particle size of nanoformulations facilitates dermal penetration by allowing passage through narrow skin pores via simple or facilitated diffusion [67]. The functional group modifications on the nanoparticle surface improve bioadhesion and promote binding to the skin, thereby extending retention time relative to conventional formulations [67]. Besides, controlled drug release from nanoformulations helps maintain a concentration gradient, improving drug deposition in the skin at any time after application (Karnam, 2023).

Skin irritancy is another critical consideration in dermal drug delivery, as it may lead to misinterpretation of treatment efficacy or reduce patient compliance due to delayed symptom resolution. Many topical antimicrobials demonstrate dose-dependent irritancy, and dose dumping is more likely with conventional formulations [66]. In contrast, nanoformulations can achieve comparable local antibacterial effects at reduced drug doses, thereby potentially minimising adverse effects [67]. While improved antibacterial efficacy remains a primary goal, ensuring biocompatibility at the application site carries the same precedence. For example, cationic lipids often show superior antibacterial activity compared to anionic or neutral lipids but are also associated with increased cytotoxicity, contributing to cutaneous irritation [68]. Similarly, chemical permeation enhancers (CPEs), though effective at promoting skin penetration, frequently cause local irritation even at their minimum effective concentrations due to their harsh chemical nature [69]. Furthermore, the alkaline shift in skin pH observed during SSTIs underscores the importance of formulating nanoformulations within the physiologically compatible pH range of 4.0 to 6.5, to avoid further irritation or breaches to the skin’s natural barrier [70].

The successful development of topical nanoformulations thus requires a careful balance between permeation, retention, and irritation. Comprehensive studies evaluating skin penetration, drug deposition, and dermal irritation are essential to substantiate the potential of these advanced systems as effective alternatives to conventional topical antimicrobials. By optimising the aforementioned parameters, topical nanoformulations yield significant advantages in terms of enhanced antimicrobial efficacy and safety. These benefits include improved drug solubility, stability, permeability, retention, and biocompatibility across dermal layers [67]. Beyond serving as carriers, some nanoformulations also possess inherent antibacterial properties, achieved through various mechanisms such as reactive oxygen species (ROS) generation, direct cell wall disruption, biofilm eradication, and interference with membrane integrity or intracellular processes which will be the main focus of this review.

## Antibacterial activity of topical nanoformulations

**Fig. 4:**
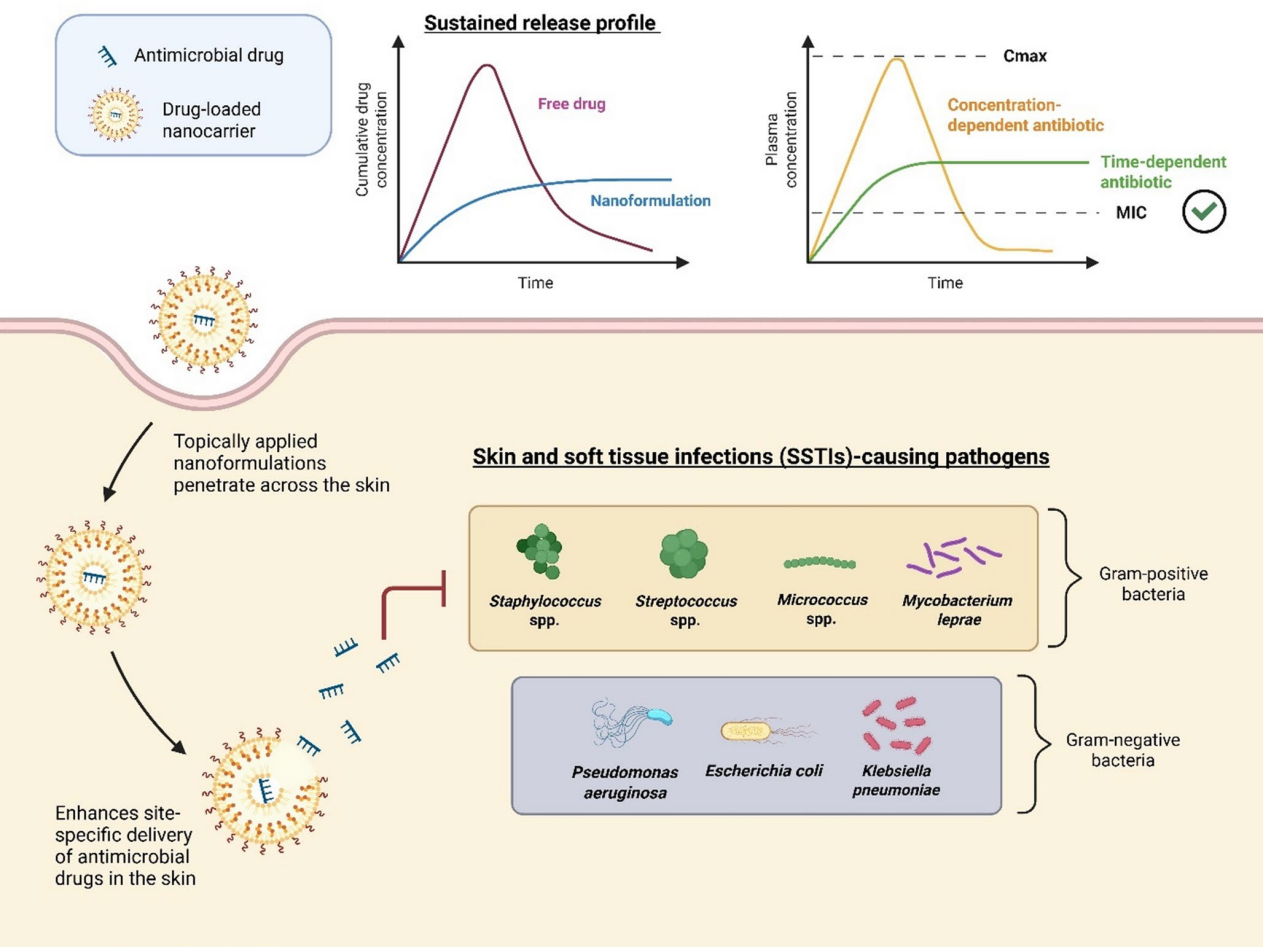
Overview of Topical Dermal Delivery of Nanoformulations against SSTI-causing Pathogens. Upon topical dermal application, the nanoformulation penetrates the skin tissue through tiny pores, delivering the drug-loaded nanocarriers directly to the site of infection and optimising the pharmacokinetic-pharmacodynamic (PK-PD) parameters against both Gram-postive and Gram-negative bacteria responsible for SSTIs

**Fig. 5:**
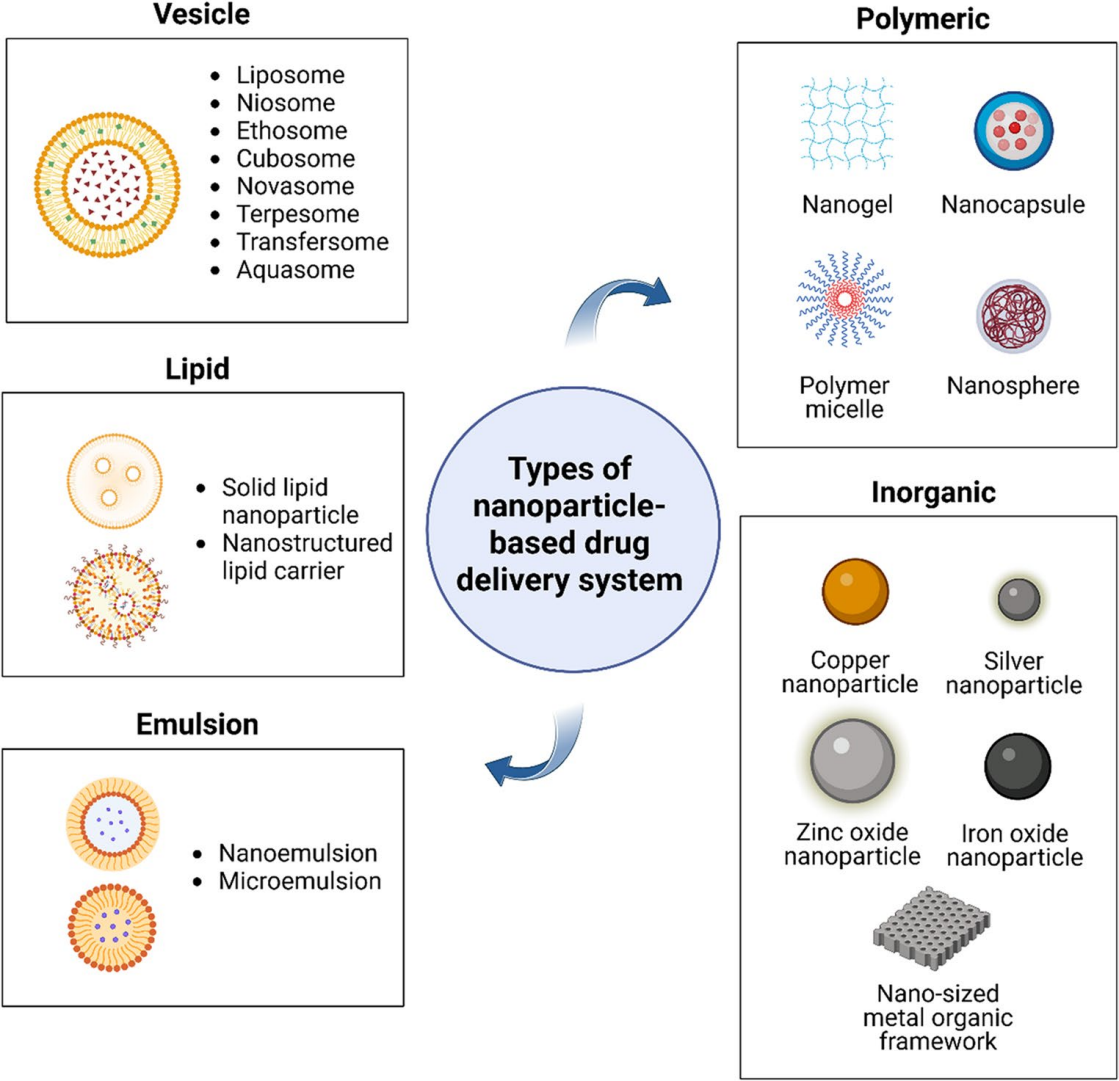
Summary of types of nanoparticle-based drug delivery system

Nanoparticle-based systems have emerged at the forefront, offering advanced, smart topical dermal delivery platforms that enhance therapeutic efficiency and help preserve or enhance the pharmacological activity of antimicrobial agents [13, 16, 71]. It is expected that nanoparticle-based topical drug delivery will offer sustained drug release, improved skin permeability and retention, additional anti-biofilm activity and promising biocompatibility, eventually fostering better therapeutic outcomes as compared to conventional topical formulations in treating SSTIs as depicted in Fig. 4 [71]. Figure 5 summarises the types of nanocarrier for topical dermal drug delivery mentioned in this review.

### Metal nanoparticles

Inorganic metal or metal oxide nanoparticles are commonplace in many different areas of pharmaceutical and biomedical research [72]. Among this group, silver, zinc oxide, copper, copper oxide and iron oxide nanoparticles represent widely studied antibacterial agents across various settings, particularly silver nanoparticles (AgNPs) in the context of SSTIs [73]. Nanoparticle formulations exhibit antimicrobial efficacy at reduced metal precursor concentrations, thereby enhancing biocompatibility. This potent activity arises from their intrinsic surface characteristics including reactive metallic elements, high surface areatovolume ratios, excellent colloidal dispersion, and structural stability, which together facilitate robust interactions with bacterial membranes [72]. Herein, metal and metaloxide nanoparticles not only serve as versatile drug delivery vehicles but also hold promise as standalone therapeutic agents due to their inherent antibacterial properties (Table 1).

**Table 1:** Summary of studies on in vitro antibacterial activity of metal nanoparticles and nMOFs

Type of NP	Active ingredient	Hydrodynamic diameter (nm)	Zeta potential (mV)	EE (%)	Bacteria	In vitro efficacyfindings	Cytotoxicity findings	Reference
AgNP	-	-	-	-	*M. sedentarius* (DMST 9365)	• Ag^0^BC inhibits bacterial growth. No antimicrobial activity with BC alone	1700 µg/mL of AgNO_3_ solution was toxic to HDFn (C-004–5 C)	[77]
AgNP	-	10 to 30 ^b^	-	-	MRSA (ATCC 14458)	• MIC of allicin and AgNPs were 2.2 mg/mL and 5.6 mg/mL, respectively • MBC of allicin and AgNPs were 3.1 ppm and 7.5 ppm, respectively • MIC and MBC of allicin and AgNPs together on MRSA strains were 0.4 mg/mL (*P* < 0.01) and 1.1 ppm (*P* < 0.01), respectively	-	[74]
Chitosan silver nanocomposite (CSN) films	-	-	-	-	*P. aeruginosa* (ATCC 9721)	• Greatest ZOI of 27.60 ± 0.26 mm with CS5 against *P. aeruginosa*; Lowest ZOI of 17.67 ± 0.40 mm with CS8 comparable to positive controls	-	[79]
ZnONP	-	10 to 50 ^b^	-	-	*S. aureus*, (ATCC 25923 and ATCC 29213), *P. aeruginosa (*ATCC 27853)	• ZOI against *S. aureus* with antibiotic alone vs. antibiotic with ZnONPs showed 28 to 44% increase; Erythromycin, 14 vs. 18 mm; Ampicillin, 12 vs. 16 mm; Vancomycin, 9 vs. 13 mm • No significant reduction in CFU of *S. aureus* between ZnONPs and vancomycin solution but to positive control (bacterial culture + PBS) • MIC of ZnONPs against *P. aeruginosa* was 32 µg/mL	No cytotoxic effects (cell viability above 80%) on PBMCs even at ZnONPs doses higher than MIC value (100 µg/mL)	[83]
ZnONP	-	49.9 ^c^	-	-	*P. aeruginosa* (ATCC 27853)	• Bacterial growth inhibition studies at 24 h, revealed 8.34 ± 0.14%, 13.72 ± 0.58%, 25.42 ± 2.19%, 41.96 ± 3.85%, 65.73 ± 3.88% and 84.62 ± 3.06% inhibition for 5, 10, 25, 50, 100 and 150 µg/mL ZnONPs, respectively.	-	[84]
CuNP	Mupirocin	413.0 ± 30.2 ^b^	-	65.2	*S. aureus* ^*^	• ZOI for *S. aureus* with mupirocin CuNP at concentrations of 0.5, 1, 4, 20, 30 and 40 µg/mL were 6 ± 2, 9 ± 2, 13 ± 3, 17 ± 1, 18 ± 2 and 18 ± 1 mm, respectively • ZOI with pure mupirocin at the same concentration ranges were 9 ± 1, 12 ± 2, 17 ± 1, 18 ± 3, 18 ± 2 and 19 ± 2 mm, respectively	-	[87]
CuONP	-	81.23 ± 2.88 ^c^	−3.50	-	*S. aureus* (MTCC 3160), *P. aeruginosa* (MTCC 358), *S. pyogenes* (MTCC 102)	• ZOI for *S. aureus*, *P. aeruginosa*, *S. pyogenes* with CuONPs were 6.7, 8.5 and 8.0 mm respectively. • ZOI for *S. aureus*, *P. aeruginosa*, *S. pyogenes* with copper acetate were 3.3, 6.0 and 5.0 mm respectively. • ZOI for *S. aureus*, *P. aeruginosa*, *S. pyogenes* with bark extract were 4.1, 6.5 and 6.5 mm respectively.	-	[88]
Magnetite-Buforin-II-silver nanobioconjugates	-	443 ± 4.5 ^a, b^	-	-	MRSA (clinical isolates: 92250621, 93040389 and 93190573)	• MIC was 2 to 32 times lower than commercial product control containing FA (20 mg/mL) (*P* < 0.0001)	Nanobioconjugates were highly cytotoxic against HaCaT (ATCC^®^ CRL-2404) cells at concentrations above 1.5% (v/v)	[92]
Photocrosslinkable nanocomposite hydrogels	Nafcillin	-	-	18.7 to 24.8	*S. aureus* (ATCC 6538)	• ZOI was greater than 29 mm, comparable to chloramphenicol (positive control) • Time-kill assay: Number of CFU after 1 h and 24 h were 1.6 × 10^5^ and 10^6,^ respectively with nanocomposite hydrogels compared to control were 2.2 × 10^8^ and 4.2 × 10^8^, respectively	-	[97]
Nanofiber	Vancomycin	201 ± 67^c^	-	63 ± 10	MRSA (clinical isolates)	• First incubation period, ZOI in the vancomycin-loaded nanofiber group were 12.1 ± 1.5, 13.5 ± 1.9 and 13 ± 1.2 mm against 3 different MRSA strains, respectively • After second incubation of the same plate, the ZOI were 6.3 ± 1.3, 3.8 ± 0.5 and 3.3 ± 0.5 mm against 3 different MRSA strains, respectively	No significant reduction in cell viability against NIH-3T3 cells (*P* > 0.05)	[98]
DNC	-	38.9 ± 1.0 ^a^	−49.4 to − 31.6	-	MRSA (clinical isolates)	• No colonies found on the agar plates of the DNC3 group after 12 and 24 h of incubation; initial bacterial count was 1.0 × 10^7^ CFU/mL • DNC1 and DNC2 exhibited a slight inhibition compared to NCC • Decreased pH of bacterial medium with DNC and NCC for 12 and 24 h, indicating pH-dependent antibacterial activity • DNC3 increased ZOI to control (0.89 ± 0.02 μm vs. 0.43 ± 0.03 μm)	Cell viability of both NIH-3T3s and HUVECs were higher than 80% compared to negative control	[99]
EPL-*g*-butyl@AgNPs	-	17.8 ^a^	-	-	*S. aureus* (ATCC 6538), *P. aeruginosa* (ATCC9027)	• ZOI for *S. aureus* was 16.74 mm compared to EPL-*g*-butyl and AgNPs, 9.06 mm and 9.1 mm respectively • ZOI for *P. aeruginosa* was 16.6 mm compared to EPL-*g*-butyl and AgNPs, 9.05 mm and 9.1 mm respectively • FIC index for *S. aureus* and *P. aeruginosa* were 0.2 and 0.1 respectively • Following 30 consecutive passages, significant increase in the control group with levofloxacin against *S. aureus* (from 0.64 to 78 µg/mL) and *P. aeruginosa* (from 3.2 to 156 µg/mL) • No difference in MIC values with EPL-*g*-butyl@AgNPs after 30 consecutive passages of both bacteria	Cell viability of NIH-3T3 cells was maintained above 80% after 2 days treatment with EPL-*g*-butyl@AgNPs at 56 µg/mL, whereas AgNPs at 56 µg/mL reduced cell viability to approximately 60%	[100]
ZnO/Gypsum/Gelatine nanocomposites films	-	40 ^c^	-	-	*S. aureus* (ATCC 25923), MRSA (ATCC 43300)	• 50 µg/mL of NCs lowered the growth of *S. aureus* by 3.5 logs (CFU/mL) compared to the untreated control (*P* < 0.01). • 50 µg/mL of NCs lowered the growth of MRSA by 3 logs (CFU/mL) compared to the untreated control (*P* < 0.001).	Percentage viability of both RAW 264.7 and 3T3 cell lines were greater than 80% at tested concentrations of 10, 30 and 50 µg/mL of NCs	[101]

Abbreviations: Bacterial cellulose, BC; Colony-forming unit, CFU; Copper nanoparticle, CuNP; Copper oxide nanoparticle, CuONP; Dialdehyde nanocrystalline cellulose, DNC; ε-polylysine/silver nanoparticle nanocomposite, EPL-g-butyl@AgNPs; Embryonic mouse fibroblast, NIH-3T3; Encapsulation efficiency, EE; Fractional inhibitory concentration, FIC; Fusidic acid, FA; Human keratinocyte, HaCaT; Human Neonatal Dermal Fibroblasts, HDFn; Human umbilical vein endothelial cells, HUVEC; Methicillin-resistant *Staphylococcus aureus*, MRSA; *Micrococcus sedentarious*, *M. sedentarius*; Minimum bactericidal concentration, MBC; Minimum inhibitory concentration, MIC; Nanocomposites, NCs; Nanocrystalline cellulose, NCC; Nanoparticle, NP; Peripheral Blood Mononuclear Cells, PBMC; *Pseudomonas aeruginosa*, *P. aeruginosa*; Silvernano bacterial cellulose, Ag^0^BC; Silver nanoparticle, AgNP; *Staphylococcus aureus*, *S. aureus*; *Streptococcus pyogenes*, *S. pyogenes*; Zinc oxide nanoparticle, ZnONP; Zone of inhibition, ZOI

^a^ Size measured using dynamic light scattering (DLS)

^b^ Size measured using transmission electron microscopy (TEM)

^c^ Size measured using scanning electron microscopy (SEM)

^*^ Isolate used unspecified

#### Silver nanoparticles

AgNPs are a form of elemental silver existing with particle sizes ranging from 10 to 30 nm [74]. Silver is widely known for its antibacterial efficacy and being incorporated into many types of wound dressings and marketed topical products, such as Aquacel Ag^®^ and Quench^®^ cream. The primary mechanism of action of AgNPs involves reactive oxygen species (ROS) generation, cell wall leakage, modulation in intracellular signal transduction, destabilization of 30 S ribosomal subunit, interruption in DNA replication and protein synthesis, leading to membrane damage [74, 75]. Silver sulfadiazine (SSD), a sulphonamide antibiotic, has been the topical agent of choice for burn-wound prophylaxis and therapy since US Food and Drug Administration (FDA) approval in 1973, owing to its accessibility, affordability, and broadspectrum activity. However, SSD has been shown to delay reepithelialisation [76]. Hence, recent advances increasingly favour AgNPs as nanocarrier delivery systems for wound management, by virtue of their superior biocompatibility in pre-clinical studies.

*Kytococcus sedentarious* is a Gram-positive bacterium that constitutes the normal skin flora, however it is duplicitously also known as one of the common causative pathogens for pitted keratolysis. Application of conventional antibiotic cream or ointment can be problematic for activities of daily living because of difficulty retaining the drug at the site of action at a highly mobile area. The rising concern emphasises the need for customising foot treatment to promote convenience while preserving treatment efficacy. The efficacy of AgNPs against *Kytococcus sedentarious* (DMST 9365) was studied by Sitlaothaworn et al. who observed zone of inhibition (ZOI) of 12 mm and 30 mm in diameter around the 6 mm and 15 mm silvernano pellicles, respectively [77]. The researchers reported absence of bacterial growth in any shoe samples within 8 to 14 days after application of silver nanoparticle-impregnated bacterial cellulose (Ag^0^BC) composites [77]. However, it remains unclear whether the nanoparticle formulation offers superior efficacy against *Kytococcus sedentarious* because the study lacked a direct comparison with silver nitrate solution.

Methicillinresistant *S. aureus* (MRSA) is a Grampositive pathogen frequently responsible for nosocomial infections as well as communityacquired SSTIs, and its incidence is particularly high in areas with elevated MRSA prevalence. The pronounced antibiotic resistance of MRSA has driven a shift in prescribing practices toward the use of broaderspectrum antibiotics or combination treatment regimens. Sharifi-Rad et al. observed complete MRSA (ATCC 14458) eradication on infected mice treated topically with AgNPs and allicin combination while the allicin treated group had 4.3 × 10^6^ CFU/mL remaining [74]. Findings from those studies suggest the combination therapy with AgNPs potentially augments the antibacterial efficacy of natural compounds such as allicin. The authors suggested a probable mechanism of increase in anti-quorum sensing and antibiofilm activity based on previous reports about AgNPs and garlic extracts individually, yet the clear mechanism of combinatorial treatment remains to be determined [74].

Evidence of AgNPs’ efficacy against Gram-negative bacteria such as *P. aeruginosa* has also emerged in recent years [78]. *P. aeruginosa*, the rod-shaped, Gram-negative bacteria that primarily causes hospital-acquired infections, is known to be highly resistant to a multitude of antibiotics and poses a looming threat to current healthcare. Researchers used chitosan as a stabiliser to fabricate chitosan-AgNP conjugates, known as chitosan silver nanocomposite films (CSN). It was found that the CSN produced the greatest ZOI of 27.60 ± 0.26 mm against *P. aeruginosa* (ATCC 9721) compared to Aquacel Ag^®^ (1.2% silver impregnated dressing) as a positive control with a ZOI of 17.67 ± 0.60 mm (*P* < 0.05) [79]. The findings suggest the superiority of the formulated CSN over the marketed AgNP-containing product, attributed to the porous film structure of CSN that triggers AgNPs release in the presence of water in addition to the inherent antimicrobial activity of chitosan [79].

#### Zinc oxide nanoparticles

Zinc oxide is another inorganic compound which acts as a broad-spectrum and photoconductive antibacterial agent [80]. One of the primary mechanisms of action is through ROS production i.e. hydrogen peroxide and superoxide anion as well as zinc ion generation that interferes with bacterial membrane proteins, DNA, and lipids, inducing oxidative stress and consequently causing cell death [80, 81]. Zinc oxide nanoparticles (ZnONPs) have a relatively small particle size (10 to 50 nm), which contributes to an increased surface area-to-volume ratio, leading to greater interactions within the bacterial microenvironment [80, 81]. This causes greater release of hydrogen peroxide, which contributes to the extensive use of ZnONPs as antimicrobials in various fields of study [82].

Use of ZnONPs in the setting of SSTIs was trialled in one study by Rauf et al. who investigated the antibacterial activity against several common SSTI pathogens. In this study, they found the MIC of ZnONPs against *S. aureus* (ATCC 25923, ATCC 29213), and *Staphylococcus epidermidis* (*S. epidermidis*) to be 32 µg/mL and 128 µg/mL, respectively [83]. From the same study, ZnONPs displayed an MIC of 32 µg/mL against *P. aeruginosa* (ATCC 27853), another common SSTI bacteria [83]. The authors also tested the combination of ZnONPs with other marketed antibiotics including erythromycin, ampicillin and vancomycin, resulting in significant ZOI of *S. aureus* treated with ZnONPs (28-to-44%-fold increase) as compared to antibiotics only [83]. A remarkable fall in the hydrophobicity index of *S. aureus* was observed after treatment with ZnONPs [83]. This suggests that ZnONPs can interrupt biofilm adherence which prevents the bacteria escaping from phagocytosis by immune cells and other lines of defence.

Dhanasegaran et al. discovered a dose-dependent pattern of ZnONPs in *P. aeruginosa* (ATCC 27853) growth inhibition using both broth microdilution and colony counting method [84]. Unfortunately, in vivo antibacterial efficacy of topical nano drug delivery against *P. aeruginosa* is yet to be established as there are currently no existing studies available. This might be due to the lack of evidence from in vitro studies which limits the progress to in vivo tests.

#### Copper nanoparticles

Copper nanoparticles (CuNPs) are used extensively in the field of medicine with applications ranging from therapeutics, diagnostic imaging, biomarker sensors, and tissue regeneration [85]. Specifically, CuNPs are incorporated as coatings to a plethora of biomedical devices (i.e. surgical tools, catheters, and implants) to prevent medical device-associated infections by leveraging its potent antimicrobial activity [85]. The release of copper ions disrupts the membrane integrity and activates the generation of ROS which causes a bactericidal effect associated with the oxidative stress response [86].

In connection with SSTIs, one study by Verma et al. et al. reported non-inferior antibacterial activity against *S. aureus* when comparing CuNPs with a well-recognised marketed antibiotic, mupirocin. Mupirocin release from CuNPs followed the Korsmeyer–Peppas model most closely, with an R^2^ value of 0.980, indicating a controlled drugrelease profile. At concentration ranges from 20 to 40 µg/mL, mupirocin coupled with CuNPs exhibited a ZOI of approximately 18 mm, comparable to that of mupirocin alone [87]. A greater difference in in vivo antibacterial efficacy is likely to be expected thanks to the controlled topical release of mupirocin on skin models. However, in vivo antibacterial test was not covered by this study.

Narayanan et al. tested the in vitro antibacterial activity of developed copper oxide nanoparticles (CuONPs) from the combination of copper acetate solution and *Thespesia populnea* aqueous bark extract, which are well-known for their antibacterial properties against *S. aureus* (MTCC 3160), *P. aeruginosa* (MTCC 358) and *S. pyogenes* (MTCC 102) [88]. The reported ZOIs with CuONPs against 3 different bacteria were approximately 1.5-fold higher than the copper acetate and bark extract alone [88]. The highest ZOI of 8.5 mm with CuONPs against *P. aeruginosa* was comparable to the standard antibiotic, chloramphenicol (Cm) 10 µg/ml (9.0 mm) [88]. Small particle sizes of CuONPs enables them to penetrate the bacterial cell wall more efficiently compared to the individual raw pharmacologically active compounds [88]. The improved penetration of CuONPs across the biological barrier reflects their potential as a topical dermal treatment for SSTIs with outstanding antibacterial activity against both Gram-positive and Gram-negative bacteria. Furthermore, the idea of leveraging antibacterial efficacy of naturally occurring bioactive compounds from plant origins by practising green synthesis of metal or metal oxide nanoparticles is supported by the findings of the study.

#### Iron oxide nanoparticles

Iron oxide nanoparticles (IONPs) are renowned for their therapeutic role in cancer, neurodegenerative conditions, iron-deficiency anaemia, last but not least, infectious diseases [89]. Similar to other metal or metal oxide nanoparticles, the underlying antibacterial mechanism of IONPs involves ROS production causing an increase in oxidative stress and damages to the bacterial biomolecules [90]. Additionally, IONPs exhibit noteworthy anti-biofilm activity, adding value to the eradication of SSTI-causing pathogens via biofilm penetration [91].

Buforin-II is a frog-derived antimicrobial peptide that can disrupt bacterial DNA replication via membrane translocation [92]. However, poor stability of the peptide under the physiological conditions deters its clinical application [92]. Munoz et al. obtained a MIC with magnetite-Buforin-II-silver (MNP-PEG8-BUF-II-Ag) nanobioconjugates dispersed in an emulsion, 2 to 32 times lower than commercial control containing FA against clinical isolates of MRSA (*P* < 0.0001) [92]. Results suggest incorporation of antimicrobial peptides to IONPs can improve their stability and enhance antimicrobial activity due to additive inherent antibacterial properties of IONPs. In the ex vivo infected porcine skin, the emulsion diffused along the skin tissue after 24 h of topical application, indicating potential on-site treatment within the dermal tissue [92]. The 3D bioprinted human skin model appears to be a promising alternative over animal models to epitomise the human skin and provide more reliable evidence for clinical translation in future. It is also highly encouraged to extend the studies with anti-biofilm assays because majority of the causative SSTI pathogens are biofilm-forming bacteria.

#### Metal organic frameworks

Metal organic frameworks (MOFs) are a class of crystalline hybrid compounds consisting of linkages between an inorganic element i.e. metal ions and organic ligands that constitutes the structure of porous, uniform, and three-dimensional lattices [93]. Through the use of nano-sized MOFs (nMOFs), multi-rate drug delivery becomes a possibility by tailoring pore sizes according to the drug molecular sizes [94]. This enables a staged delivery mechanism to maximise the potential of antimicrobial agents which possess different pharmacokinetic-pharmacodynamic (PK-PD) parameters, which is especially useful in combinatorial therapy. Pertaining to the delivery of antibacterial agents, use of zeolites, activated carbon, and mesoporous silica have been broadly investigated [95]. The acceptable cytotoxicity of nMOFs has been proposed to be as low as the other commercialised nanosystems which provides solution to the drawback of metal or metal oxide nanoparticles [93, 96].

One study by Toader et al. reported reduction in bacterial growth after 1 h of a time-kill assay (2.6 × 10^5^ CFU/mL and 5.6 × 10^6^ CFU/mL) with nafcillin-loaded titanium dioxide (TiO_2_) nanocomposites and ZnO nanocomposites, respectively, compared to the blank reference strains (2.2 × 10^8^ CFU/mL), indicating that the formulations possess outstanding antibacterial efficacy [70]. TiO_2_ nanocomposites with larger pores and dimensions of titanium dioxide nanoparticles (TiO_2_NPs) manifested faster nafcillin release in the first 2 h compared to ZnO nanocomposites [70]. This is due to the larger surface area of ZnO nanocomposites ascribed to the smaller dimensions of ZnONPs that promote stronger interactions between ZnONPs and nafcillin. Findings support the use of assorted nanofillers (e.g. TiO_2_ and ZnO), to modify the pore characteristics and customise the rate of drug delivery based on their ideal PK-PD parameters.

Another study by Fathi et al. evaluated the sustained antibacterial effect of vancomycin-loaded nanofibers against clinical isolates of MRSA. After 2 consecutive 24-hour incubations, there was no inhibition observed in the control group compared to a ZOI of 3.3 to 6.3 mm obtained in the group treated with the nanofibers [97]. Vancomycin is a glycopeptide which exhibits time-dependent bacterial killing. Sustained drug release from nanofibers as evidenced in the repeated incubation for the ZOI study increases the exposure of MRSA to vancomycin over a prolonged period that translates to improved antibacterial activity by incorporating the time-dependent antibiotics into nanofibers. Findings imply that the sustained release profile of nMOFs is a key advantage as nanocarrier to enable the full efficacy potential of time-dependent antibiotics and minimise the dose-dependent adverse effects by lowering the peak concentration (Cmax) which is a vital indicator of safety.

SSTIs can be classified into superficial infections and deep dermal infections where topical formulations may differ in antibacterial efficacy due to their distinctive penetration, dependent on the thickness of different skin layers. Luo et al. established MRSA-infected mice models to compare the therapeutic efficacy of dialdehyde nanocrystalline cellulose (DNC) applied topically on normal intact skin (NS), injured skin (IS) and colonised skin (SC) [98]. MRSA invasion can cause deep dermal infection across the compromised skin barrier as seen in IS group. There was no significant statistical difference in the number of CFU following topical treatment with DNC or gentamicin sulphate (positive control) [98]. DNC was found to provide wound healing benefits and can be considered as an alternative to the marketed antibiotic, gentamicin sulphate used topically for better therapeutic outcome [98].

In another study, researchers investigated the ability of bacteria to develop resistance against a benign crystalline ε-polylysine/silver nanoparticle nanocomposite (EPL-*g*-butyl@AgNPs) compared to levofloxacin. Inherently, AgNPs are less likely to trigger development of bacterial resistance by targeting multiple biological processes with their antibacterial mechanisms, however, the process of nanoparticle aggregation results in loss of antimicrobial activity due to decrease in total surface area-to-volume ratio [99]. Incorporating AgNPs into a crystalline cubic structure enables the formation of nMOFs, which helps maintain a high surface areatovolume ratio and preserve the antimicrobial efficacy of silver during nanoparticle formation. A recent study also showed significant bacterial reduction of *S. aureus* (*P* < 0.01) and MRSA (*P* < 0.001) with ZnONP/Gypsum/Gelatine nanocomposite film compared to the untreated control [100]. These findings highlight the potential of nMOFs as a promising enhancement to metal nanoparticles for the topical treatment of SSTIs.

### Emulsion-based nanosystems

Emulsions-based nanocarrier systems are nanometric, biphasic, colloidal dispersions made up of two immiscible liquids stabilised by an interfacial surfactant system, also known as emulsifiers [101–107]. Emulsions can be classified into 3 categories: oil-in-water (o/w), water-in-oil (w/o), and bi-continuous, based on the internal phase ratio and hydrophilic-lipophilic balance (HLB) of the surfactant system and internal phase [105, 106]. In o/w emulsions, oil is the dispersed phase (internal phase) and water is the continuous phase (external phase) which can serve to encapsulate hydrophilic drugs, whereas in w/o emulsions, the opposite is true, enabling the system to carry water-insoluble drugs [103, 105, 106]. Bi-continuous refers to the phase inversion from o/w to w/o and vice versa when salt is added, or due to an increase in internal phase ratio that disrupts the balance of surfactant system. Emulsions-based nanocarrier systems are examined thoroughly for their potential use in topical dermal drug delivery for the treatment of SSTIs (Table 2).

**Table 2:** Summary of studies on in vitro cytotoxicity and antibacterial activity of emulsion-based nanosystems

Type of NP	Active ingredient	Hydrodynamic diameter (nm)	Zeta potential (mV)	EE (%)	Bacteria	In vitro efficacy findings	Cytotoxicity findings	Reference
Microemulsion	Azithromycin	94 to 138 ^a^	−22.32 to − 32.60	-^+^	MRSA (clinical isolates: #7 and #83), *S. aureus* (ATCC 29213)	• Unloaded microemulsions did not display antibacterial activity • M1 and M2 displayed MIC values of 4 µg/mL and 2 µg/mL, respectively equal to that of free AZT for MRSA strains #7 and #83 • M3 displayed an MIC of 4 µg/mL for both strains #7 and #83, equal to that of free AZT for strain #7 and two-fold to strain #83 • Both AZT-loaded microemulsions and free AZT demonstrated MICs of 1 µg/mL against *S. aureus*	• At 20 µg/mL, M1 and M3 showed cell viability above 70% against WS1. • At 40 µg/mL, all the loaded microemulsions were cytotoxic	[64]
Lipid-based microemulsion gel	Tea tree oil	14.4 ± 4.4 ^b^	-	-^+^	*S. epidermidis* (ATCC 35984), *P. aeruginosa* (PAO1)	• ZOI of *S. epidermidis* were 4.2 mm with TTO-LNF gel and 5 mm with TTO-LNF • ZOI of *P. aeruginosa* with TTO-LNF and TTO-LNF gel were 6.4 mm and 7.8 mm, respectively	-	[109]
Nanoemulsion	Kojic acid ester	110.01 ± 0.14 ^a^	-	-	*S. aureus* (ATCC 43300)	• ZOI diameter for *S. aureus* with kojic acid ester-based NE and kojic acid esters were 8.0 mm and 6.5 mm	-	[102]
Myrrh oil nanoemulgel	Fusidic Acid	113.6 ± 3.21 ^a^	-	-^+^	*S. aureus* (ATCC 29213)	• ZOI with FA-NEG, Placebo NEG and marketed FA cream were 4.4 ± 0.17, 2.2 ± 0.10, and 3.9 ± 0.15 cm, respectively	-	[101]
Nanoemulsion	Soyaethyl morpholinium ethosulfate (SME)	214.4 ± 1.6 ^b^	54.1 ± 0.1	-	MRSA^*^, S. epidermidis*, S. aureus^*^	• MIC against *S. epidermidis*, *S. aureus* and MRSA were 0.08 to 0.16 µg/mL, 1.4 µg/mL, and 2.8 to 5.6 µg/mL, respectively • NEs showed a higher degree of *S. epidermidis* death as compared with liposomes at 50 µg/ml	Cell viability of HaCaT was greater than 85% with NEs, while THP-1 and neutrophils were 60% and 57%, respectively	[104]
Chitosan nanoemulgel	Omeprazole	369.7 ± 8.77 ^a^	−15.3 ± 6.7	78.23 ± 3.76	*P. aeruginosa* ^*^	• Moderate MIC value (1.25 mg/mL) with optimized nanoemulgel formulation (OMP3NEG)	-	[107]

Abbreviations: Azithromycin, AZT; Encapsulation efficiency, EE; Fusidic acid, FA; Fusidic acid-loaded nanoemulgel, FA-NEG; Human fibroblast, WS1; Human keratinocyte, HaCaT; Macrophages, THP-1; Methicillin-resistant *Staphylococcus aureus*, MRSA; Nanoemulsion, NE; Nanoemulgel, NEG; Nanoparticle, NP; Omeprazole-loaded nanoemulgel, OMPNEG; *Pseudomonas aeruginosa*, *P. aeruginosa*; Soyaethyl morpholinium ethosulfate, SME; *Staphylococcus aureus*, *S. aureus*; *Staphylococcus epidermidis*, *S. epidermidis*; Tea tree oil lipid-based nanoformulation, TTO-LNF; Zone of inhibition, ZOI

^a^ Size measured using dynamic light scattering (DLS)

^b^ Size measured using transmission electron microscopy (TEM)

^*^ Isolate used unspecified

^+^ Data not reported

#### Microemulsions

Microemulsions are isotropic, monodisperse droplets sized about 10 nm formed spontaneously in an exact mixture ratio of two immiscible phases and surfactants without any external energy input [105]. Microemulsions are thermodynamically stable indefinitely in nature, provided physical variables such as internal phase volume ratio, temperature, and pressure remain constant [105]. A relatively higher concentration of surfactants and co-surfactants are required for the production of microemulsions to diminish the free interphase energy of the system at their lowest energy state which may induce a safety concern [106]. An example of a microemulsion-based commercial product is a gel containing lidocaine, Topicaine^®^ (ESBA Laboratories Inc., Jupiter, FL, USA) used for localised pain relief which evidences the role of microemulsions in transporting hydrophobic agents via a topical route [108].

Abruzzo et al. stated that lower interfacial tension of microemulsions could be attributed to their thermodynamic stability, eventually resulting in very small particle sizes [64]. The authors disclosed significantly greater accumulation of azithromycin (AZT), 59.58 ± 3.49% in an ex vivo porcine ear full-thickness skin model 24 h post-application compared to the control, 22.04 ± 3.99% retained in the skin *(P* < 0.05*)* [64]. Owing to the intrinsic negative surface charge of the skin lipids, AZT-loaded microemulsions exhibiting a negative zeta potential, typically ranging from − 22.32 to − 27.78 mV demonstrated enhanced skin penetration, outperforming their positively charged or neutral counterparts in dermal drug delivery applications [64]. This suggests that microemulsions can be considered as a suitable alternative by addressing the barrier issue to develop topical nanoformulations for treating SSTIs.

Muta et al. compared a tea tree oil lipid-based nanoformulation gel (TTO-LNF gel) and TTO-LNF, a microemulsion against *P. aeruginosa* (PAO1) which observed a ZOI of 7.8 mm and 6.4 mm, respectively [109]. Moreover, the TTO-LNF gel was found to be more effective in inhibiting the growth of *P. aeruginosa* than the 5% TTO solution [109]. This is in contrast to *S. epidermidis* (ATCC 35984), with a ZOI of 4.2 mm for TTO-LNF gel and 5 mm for TTO-LNF [109]. The thicker peptidoglycan layer of Grampositive bacteria may act as a protective barrier, impeding the penetration of TTOLNF gel. Uncertainties remain on whether the developed TTO-LNF gel exceptionally improves the antibacterial activity against Gram-negative bacteria.

#### Nanoemulsions

Nanoemulsions exist in liquid droplet forms, approximately 100 nm in size [105]. In fact, the formation of nanoemulsions does not occur spontaneously but requires high-energy emulsification methods such as high-pressure homogenisation, ultrasonic emulsification, high-energy stirring, microfluidisation and membrane emulsification [103, 105, 106]. The application of high external energy input generates kinetically stable nanoemulsions that resist phase separation over time, despite not being in a thermodynamic equilibrium state. Theoretically, nanoemulsions exhibit better biocompatibility due to their relatively low surfactant concentration compared to microemulsions [103, 105, 106]. Notable examples of nanoemulsion-based commercial products include Ameluz^®^ topical gel (Biofrontera Pharma GmbH, Leverkusen, Germany) containing aminolevulinic acid for actinic keratosis, and NanoCacao Mibelle Biochemistry containing cocoa beans for antiaging purposes [106, 108].

As an extension, nanoemulsions can be used in topical treatment of dermatological conditions such as atopic dermatitis and SSTIs, owing to their capacity to enhance skin penetration and improve drug solubility evidenced by their widespread use in the current formulation industry [110]. Azhar et al. reported a ZOI diameter of *S. aureus* (ATCC 43300) treated with kojic acid ester encapsulated nanoemulsion and kojic acid ester alone to be 8 mm and 6.5 mm, respectively [102]. Kojic acid ester is poorly soluble in water due to the ester functional group and is also susceptible to enzymatic degradation. The authors proposed the encapsulation of kojic acid ester within a nanoemulsion improved its stability and water solubility thus enhancing its antibacterial efficacy in topical application [102]. A study by Almostafa et al. compared fusidic acid-loaded myrrh oil nanoemulgel (FA-NEG) and marketed FA cream against *S. aureus* (ATCC 29213) [101]. FA has low water solubility, thus incorporation into a myrrh oil nanoemulgel to overcome this weakness enables its application in topical therapy. Nanoemulgel enhances the dissolution of FA within the oil-water interface and facilitates intracellular drug transport across the bacterial cell wall and membrane [101]. Myrrh oil also exhibits innate antibacterial activity in addition to FA which further improves the antibacterial efficacy.

In another study by Lin et al. which tested the antibacterial efficacy of nanoemulsions, and liposomes intercalated with soyaethyl morpholinium ethosulfate (SME) in an oil-water interface and a phospholipid bilayer, respectively. SME is a cationic, amphiphilic surfactant which possesses inherent antibacterial activity via interaction with the negatively charged bacterial surfaces, disrupting the membrane integrity and permeability, consequently, leading to cell death [104]. The authors obtained a significant reduction in MRSA burden in both nanoemulsion- and liposome-treated groups compared to the untreated group (*P* < 0.05) using an in vivo infected mice model [104]. Nevertheless, the nanoemulsion-treated group had greater reduction in CFU than the liposome-treated group (> 200-fold) [104]. The presence of the oil–water interface enabled greater intercalation of SME on the surface of nanoemulsions with larger particle size and smaller particle counts (214.4 ± 1.6 nm and 2.7 × 10^9^) compared to liposomes (75.0 ± 7.8 nm and 3.5 × 10^11^), resulting in a higher overall positive surface charge of the nanoemulsions (54.1 ± 0.1 mV) compared to liposomes (38.2 ± 4.2 mV) [104]. This enhanced surface charge aids in stronger bioadhesion of the nanoemulsions to bacterial cells, leading to complete membrane rupture and lysis, hence better in vivo antibacterial effect than liposomes.

Omeprazole is a proton pump inhibitor (PPI) which can potentially be repurposed for the treatment of SSTIs due to its antimicrobial effect. One study by Ullah et al. reported statistically significant reduction in MIC (*P* < 0.05) of an omeprazole-loaded nanoemulgel compared to an omeprazole-loaded nanoemulsion against *P. aeruginosa* [107]. These findings proved the advantage of nanogel systems: the incorporation of a gelling agent with increased viscosity and prolonged drug release properties potentially improves activity against multidrug-resistant Gram-negative bacteria causing SSTIs such as *P. aeruginosa*. Nevertheless, limitations of the study include poor correlation of the in vitro drug release study to the ZOI study due to the absence of sink conditions and fixed microenvironments for different treatment groups.

### Nanovesicles

Nanovesicles are nanosized spherical, core-shell, membrane-bound structures made up of amphiphiles self-orienting in an aqueous environment [111–119]. The general structure consists of a bilayer with two compartments, an aqueous core and hydrophobic region within the lipid bilayer which makes it an attractive candidate as a nanocarrier for drug delivery. It is feasible to encapsulate polar or hydrophilic agents in the aqueous core, while any non-polar or hydrophobic agents can be incorporated into the lipid bilayer via hydrophobic interactions. To date, a great deal of vesicular systems thrives in accommodating the need for effective topical dermal SSTI therapy including liposomes, niosomes, ethosomes, cubosomes, novasomes, terpesomes, nanotransfersomes and aquasomes (Table 3).

**Table 3:** Summary of studies on in vitro cytotoxicity and antibacterial activity of nanovesicles

Type of NP	Active ingredient	Hydrodynamic diameter (nm)	Zeta potential (mV)	EE (%)	Bacteria	In vitro efficacy findings	Cytotoxicity findings	Reference
Liposome	Soyaethyl morpholinium ethosulfate (SME)	75.0 ± 7.8 ^a^	38.2 ± 4.2	-	MRSA^*^, S. epidermidis*, S. aureus^*^	• MIC against *S. epidermidis*, *S. aureus* and MRSA were 2.8 µg/mL, 11.2 µg/mL, and 22.4 µg/mL, respectively	• LP had viabilities of > 85% against HaCaT, THP-1 (79%) • No significant cytotoxic effect against neutrophils (*P* > 0.05)	[104]
Liposome	Azithromycin	132 to 165 (except for AZT-loaded CATLs is 217 nm) ^a^	-40 to -50 (except for AZT-loaded CATLs is + 60)	45 to 64	MRSA (clinical isolates: MFBF 10674, MFBF 10676, MFBF 10677, MFBF 10679, MFBF 10680)	• MIC of free AZT against MRSA strains ranged between 2 to 8 µg/mL • MIC of AZT-loaded LP ranged between 0.25 to 4 µg/mL	• At 0.25 to 64 µg/mL of AZT-loaded LPs, viability of HaCaT remained above 70% • At 64 µg/mL of AZT-loaded LPs, viability of MJ90hTERT fibroblast was reduced to below 70%	[114]
Liposome	Chloramphenicol	DMPC-LP: 132.1 ± 43.6 DA-LP: 238.5 ± 17.1 ^a^	DMPC-LP: −37.8 ± 4.5 DA-LP: −39.4 ± 8.4	DMPC-LP: 14.1 ± 2.0 DA-LP: 12.5 ± 2.3	MRSA (ATCC 33591)	• MIC and MBC of DMPC-LP were 62.5–125 µg/mL and 125–250 µg/mL, respectively • MIC and MBC of DA-LP were 62.5 µg/mL and 62.5–125 µg/mL, respectively • Similar inhibition diameter between free Cm and DMPC-LP while DA-LP had about 2-fold wider • Comparable death rate (95%) of MRSA between DA-LP and free Cm using flow cytometry • No significant difference between DA-LP with Cm and free Cm in intracellular MRSA killing at 62.5 ug/mL and 125 ug/mL	• Empty LP without Cm were used • DA-LP, DMPC-LP and classic LP showed 81%, > 80% and 59% viability to HaCaT at the highest SPC concentration (1.25 mg/mL) • All LPs maintained neutrophil viability > 85% at 0.25 to 1.25 mg/mL	[113]
Niosome	Melittin	120 to 200 ^c^	−7 to − 11	> 90	*S. aureus* (ATCC 25923), MRSA (ATCC 43300), VISA (clinical isolates)	• Mellitin-loaded niosomes significantly inhibited all 3 bacterial growths in a dose-dependent manner (*P* < 0.05)	• Empty niosomes were used • No cytotoxicity on NIH-3T3 and A375 cells from 100x to 10x dilution • Significant reduction in viability to both cell lines at x2 dilution (*P* < 0.05)	[117]
Cubosome	Amoxicillin trihydrate	133.03 ± 7.45 ^c^	-	94.76 ± 0.27	*S. aureus* (ATCC)	• ZOI with AMT-loaded LLCNPs were 29 mm at about 50 µg/mL and 34 mm at 200 µg/mL concentration • ZOI with plain AMT solution were 28 mm at 50 µg /mL and 33 mm at 200 µg/mL • MIC of AMT-loaded LLCNPs was 0.13 µg/mL	• Almost 100% viability of HDF with 25 µg/mL of AMT-loaded LLCNPs • About 86% viability of HDF with 25 µg/mL of AMT solution	[112]
Novasome	Luteolin	383.4 ± 19. 7 ^b^	-7.9 ± 2.57	79.8 ± 1.9	MRSA (clinical isolates: MS3, MS15, MS16, MS17, MS23 and MS43)	• MIC of LUT dispersion was 39–1250 µg/mL • MIC of LUT-loaded novasomes was 19.5–39 µg/mL • Neither LUT dispersion nor LUT-loaded novasome showed bactericidal activity	• LUT-loaded novasomes and LUT dispersion showed IC50 > 300 µg/mL and 8.9 µg/mL to HDF, respectively	[119]
Terpesome	Levocetirizine dihydrochloride	243.30 ± 4.60 ^b^	23.20 ± 0.55	69.64 ± 0.14	MRSA (USA300)	• MIC of TPs-gel was 0.469 mg/mL	-	[111]
Aquasome	Cephalothin	452^a^	-14.6	62 ± 2	*S. aureus* ^*^	• ZOI for *S. aureus* with cephalothin-loaded aquasome was 38 ± 3 mm • ZOI for *S. aureus* with cephalotin was 29 ± 5 mm	-	[139]

Abbreviations: Amoxicillin trihydrate, AMT; Azithromycin, AZT; Cationic liposomes, CATLs; Chloramphenicol, Cm; Dimyristoylphosphatidylcholine, DMPC; Deoxycholic acid, DA; Encapsulation efficiency, EE; Liposome, LP; Luteolin, LUT; Lyotropic liquid crystalline nanoparticle, LLCNP; Human dermal fibroblast, HDF; Human malignant melanoma, A375; Methicillin-resistant *Staphylococcus aureus*, MRSA; Minimum bactericidal concentration, MBC; Minimum inhibitory concentration, MIC; Nanoparticle, NP; Normal embryonic mouse fibroblasts, NIH-3T3; Soyaethyl morpholinium ethosulfate, SME; Soybean phosphatidylcholine, SPC; *Staphylococcus aureus*, *S. aureus*; *Staphylococcus epidermidis*, *S. epidermidis*; Terpesome, TP; Vancomycin-intermediate *S. aureus*, VISA; Zone of inhibition, ZOI

^a^ Size measured using dynamic light scattering (DLS)

^b^ Size measured using transmission electron microscopy (TEM)

^c^ Size measured using scanning electron microscopy (SEM)

^*^ Isolate used unspecified

#### Liposomes

Liposomes represent one of the first pioneered vesicular drug delivery nanocarrier systems used in medical application as evidenced by numerous marketed products, such as US FDA approved Amphotec^®^ and Ambisome^®^ containing liposomal amphotericin B used prevalently for fungal infections caused by Candida spp. and Aspergillus spp.; Epaxal^®^ vaccines for Hepatitis A and Inflexal^®^ V vaccines for influenza in the form of virosomes [120, 121]. Mainly comprised of phospholipid molecules, liposomes rely on the self-assembly of its building material into a spherical configuration when placed in an aqueous environment forming the famously known bilayer structure. Addition of other constituents such as cholesterol, polymers or surfactants can alter the membrane fluidity and permeability for controlled drug delivery [113, 114, 121]. Nonetheless, limitations of conventional liposomes include short circulation half-life, potential drug leakage and poor inherent stability [118].

Rukavina et al. found that liposomal formulations produced improved MIC values of AZT against both *S. aureus* (ATCC 29213) and clinical isolates of MRSA compared to free AZT across the board, with the most effective formulations being cationic liposomes (CATLs), followed by deformable liposomes (DLs), propylene glycol-containing liposomes (PGLs) and conventional liposomes (CLs); though the least effective CLs still exhibited activity twice as high as that of free AZT [114]. Positively charged CATLs are more likely to adhere to the bacterial surface with negative charges [114]. Plus, CATLs have inherent bactericidal activity attributed to the cationic lipid dimethyldioctadecylammonium bromide (DODAB) which explains the findings in the study [114]. This study highlights the fact that antibacterial efficacy of the liposomal drug delivery systems is highly dependent on the bilayer composition and vesicular surface charge.

Theoretically, deformable liposomes remain intact and stable after skin penetration which facilitates sustained drug release that enhances anti-MRSA activity by maintaining prolonged exposure to antibiotics at concentrations above the MIC [113]. Hsu et al. reported a 2-fold wider ZOI with deoxycholic acid (DA) liposomes loaded with Cm against MRSA (ATCC 33591) compared to free Cm at 0.5 mg/mL (*P* < 0.01) [113]. However, dimyristoylphosphatidylcholine (DMPC) liposomes loaded with Cm showed comparable ZOI compared to the control. A significantly lower MRSA death rate of 73% (*P* < 0.05) was also found with Cm-loaded DMPC liposomes compared to free Cm, classic liposomes and DA liposomes [113]. DMPC liposomes have their malleable feature compromised secondary to the structural instability after penetrating the skin [113].

In connection with the previous study, further modification of classic liposomes with respect to structural and compositional parameters such as elasticity and surface potential is advisable to enhance the antimicrobial efficacy of conventional antibiotics for topical dermal treatment of SSTIs. To overcome the limitations associated with classic liposomes, deformable cationic liposomes are recommended, as they favour more efficient dermal drug delivery and improved therapeutic outcomes in SSTI management. This design strategy can be implemented through the incorporation of novel amphiphilic compounds, including synthetic lipids with polar head groups such as DODAB, DMPC, and DA, in liposomal formulations.

#### Niosomes

Niosomes, also known as non-ionic surfactant vesicles, have different structural composition from liposomes, constituting non-ionic surfactants based on their HLB values and cholesterol [122, 123]. The hydrophilic end of the non-ionic surfactant points outwards while the hydrophobic regions orient inwards to form two discrete compartments available for drug encapsulation [123]. Niosomes are widely used in cosmetic field with several marketed topical formulations such as Lancome (anti-aging), Britney Spears (Lip gloss) and Loris Azzaro (Lipcolour and lipstick). Niosomes present a suitable alternative to liposomes, particularly when drug encapsulation disrupts the phospholipid bilayer, leading to drug leakage [122, 123]. By virtue of their composition of non-ionic surfactants, niosomes offer superior formulation stability, enabling efficient drug loading without compromising the integrity of the vesicular structure which favours their application as nanocarrier in delivering topical SSTI therapy.

Sangboonruang et al. studied the activity of fabricated melittin-loaded niosomes against *S. aureus* (ATCC 25923), MRSA (ATCC 43300) and clinical isolates of vancomycin-intermediate *S. aureus* (VISA). In vitro results from the study showed significant antibacterial activity of melittin-loaded niosomes compared to a phosphate buffered saline (PBS)-treated control (*P* < 0.05) [117]. There was a reduction of *S. aureus* bacterial CFU from 2.64 × 10^10^ to 1.75 × 10^10^ CFU/mL/g tissue for intact ex vivo porcine ear skin after 4 h of melittin-loaded niosome topical treatment [117]. A vital limitation in this work is that the study only drew comparisons between the treated group with melittin-loaded niosome and untreated group with PBS which did not clearly demonstrate the significance of encapsulating melittin into niosomes due to insufficient CFU data with melittin alone.

#### Ethosomes

Another lipid-based vesicular nanosystem, ethosomes, gain their moniker owing to their high ethanol composition (20 to 50%) [115, 118]. The unique composition of ethosomes have been proposed to potentially improve skin penetration due to their flexible characteristics. Their soft, malleable and highly flexible nature are due to the relatively high proportion of ethanol as a constituent which eases penetration across the epidermis compared to the conventional liposomes. As a result, numerous studies have employed this particular system in targeting the dermal and transdermal route [124–126]. Sahu et al. developed linezolid-loaded ethosomes who observed full recovery and hair regrowth after being treated topically with 14 days of linezolid-loaded ethosomes using a deep dermal *S. aureus*-infected rat model, followed by similar progression in both liposome and hydroethanolic linezolid solution treated groups [115]. The enhanced skin permeation granted by the flexibility of ethosomes serves as a unique advantage compared to other vesicular systems. This can potentially overcome the problem associated with topically administered liposomes accumulating in the stratum corneum [127]. The accumulation in the stratum corneum typically indicates poor penetration into the deeper skin layer which leads to subtherapeutic outcomes and progression of drug-resistant deep SSTIs.

#### Cubosomes

Cubosomes or lyotropic liquid crystalline nanoparticles (LLCNPs) are composed of amphiphilic lipids and surfactants that self-organise into a honeycombed-like, three dimensional tightly packed structures with bi-continuous cubic phases of water and lipid domains in the form of liquid crystalline particles [128]. The structural formation usually occurs in an aqueous surfactant system encompassing a high proportion of amphiphiles and polymers that act as stabilisers to the colloidal dispersion, which explains why cubosomes are generally thermodynamically stable [129]. Cubosomes differ from liposomes by its structure which allows simultaneous loading of hydrophilic, lipophilic, and amphiphilic agents in the three-phase regions containing a liposomal dispersion [128]. The structural resemblance of cubic phase to human skin layers suggests it could enhance skin permeation and drug deposition to potentiate topical dermal drug delivery [129]. By closely replicating skin architecture, cubosomes exhibits intrinsic bioadhesion to cutaneous and mucosal surfaces, making them promising candidates for dermal drug delivery systems [130].

Gitte et al. studied the antibacterial efficacy of cubosomes loaded with amoxicillin trihydrate (AMT-loaded LLCPs) against a commercial strain of *S. aureus* and reported a ZOI of 29 mm which was non-inferior to 28 mm observed with free AMT solution at 50 µg/mL [112]. Non-inferiority indicates that the cubosome drug delivery system did not negatively impact the antibacterial activity of amoxicillin in any way. Amoxicillin belongs to the antibiotic class of penicillin, a time-dependent antibiotic whereby the duration of antibiotic concentration above MIC (T > MIC) determines its antibacterial activity. Small pore size and tortuous structure of the cubic phases of cubosome tend to slow down the diffusion of solubilised antimicrobial agents for controlled release. The in vitro AMT release study revealed that AMT-loaded LLCPs released 66.72 ± 1.45% of the drug within 2 h, significantly slower (*P* < 0.05) than the free AMT solution (85.76 ± 9.87%) [112]. AMT-loaded LLCPs is likely to achieve a longer T > MIC compared to free AMT solution which implies the improved bacteriostatic action. Cubosomes have great potential as alternative nanocarriers for topical SSTI therapy due to the additional benefits and properties that can resolve the limitations of liposomes, particularly low formulation stability.

#### Novasomes

Novasomes are one of the latest nanovesicle formulations that were first established in the market with the development of an FDA-approved protein-based Covid-19 vaccine, NOVAVAX [131]. It was proposed by Mosallam et al. that the main component of novasomes is free fatty acids (FFA), commonly stearic acid in addition to the basic composition of lipid vesicles, adding value to its superior drug delivery compared to liposomes and niosomes [132]. Zakaria et al. tested luteolin-loaded novasomes against MRSA clinical isolates and reported an MIC of 19.5 to 39 µg/ml compared to 39 to 1250 µg/ml when using luteolin dispersion [119]. The authors also conducted an in vivo study using a murine model to test the topical application of luteolin-loaded novasomes compared to luteolin dispersion and a positive control, commercially available topical ointment containing 2% FA. There was statistically significant reduction in MRSA burden of the infected skin reported in the groups treated with luteolin-loaded novasomes (*P* < 0.05) and FA (*P* < 0.05), respectively compared to luteolin dispersion [119]. Luteolin is a natural-occurring antibacterial agents with limited clinical use because of its poor water solubility. Thus, novasomes as nanocarriers improves the drug solubilisation that allows it to outperform commercial topical antibiotic formulations.

#### Terpesomes

Terpesomes, or invasomes, are developed by integrating terpenes such as eugenol and limonene, phytochemicals consisting of isoprene monomers which self-assemble to form a vesicular nanoparticle system. Tawfik et al. proposed the role of terpene as a chemical permeation enhancer (CPE) to promote drug transport across the transdermal route [133]. Although CPE are easily incorporated into topical formulations, their use is often associated with biocompatibility concerns [134]. This is primarily due to their direct interference with membrane permeability and disruption of intracellular transport processes, which may elicit irritating cutaneous responses. Terpesomes, with their small lipophilic structures, are expected to traverse the narrow intercellular spaces of the stratum corneum, thereby enhancing skin penetration [111]. Additionally, the intrinsic enhancer properties of terpenes within terpesomes can be leveraged at lower concentrations than those typically required in transdermal formulations, many of which use up to 5% w/v terpenes to achieve adequate penetration. This strategy may reduce the risk of local irritation, as the irritant potential of terpenes is likely to be concentration dependent [134].

El-Naggar et al. observed higher in vivo antibacterial activity of topically applied levocetirizine-loaded terpesome gel (TPs-gel) with a reduction in MRSA (clinical isolate) burden by 3.14 (log_10_ CFU) (*P* < 0.01), while levocetirizine dihydrochloride gel (LVC-gel) had 1.62 (log_10_ CFU) (*P* < 0.01) lower than the negative control group [111]. LVC, a hydrophilic antihistamine was studied for its antibacterial activity as its R-enantiomers, cetirizine was previously found to exert antibacterial action. Both molecules shared the same molecular formula, but different spatial arrangement which suggested a potential antibacterial activity of LVC. The researchers established the improved skin permeation and sustained LVC release from terpesomes that led to enhanced in vivo antibacterial effect to agree with the results of ex vivo permeation study using a rat skin model. The application of terpesomes holds potential for topical antimicrobial agent delivery in the skin for the treatment of SSTIs as an alternative to CPEs in conventional drug delivery formulations.

#### Transfersomes

Transfersomes are bilayer vesicles composed of membrane stabilising agents, i.e. phospholipids and edge activators, i.e. single chain surfactants such as ethanol that increase the capacity of tolerance to ambient stress [135]. Transfersomes are more elastic and ultra-deformable than liposomes with the presence of edge activators destabilising the vesicles which suggests the potential role in dermal drug delivery with improved skin penetration [136]. Salatin et al. (2020) evaluated cephalexin transfersome (NT) chitosan hydrogel compared to a plain cephalexin hydrogel using an in vivo *S. aureus* infected rat skin model. After ten days of treatment, the authors reported bacterial counts in the skin treated with cephalexin NT hydrogel to be 2.95 ± 1.52% (*P* < 0.05) of the untreated control indicating almost complete recovery, while the plain cephalexin hydrogel group had 23.55% (*P* < 0.05) of the untreated control [116]. In ex vivo rat skin studies, the cephalexin NT hydrogel showed significantly greater penetration (*P* < 0.05) than plain hydrogel over 8 h [116]. The superior permeation performance of the NT hydrogel can be attributed to its enhanced deformability, underscoring their potential for optimised dermal drug delivery.

#### Aquasomes

Aquasomes are non-lipoidal, vesicular three-layered nanostructures, consisting of solid nanocrystalline cores, polyhydroxy oligomer coating and drug [137, 138]. Common inorganic materials used to prepare the cores include diamonds, tin oxide and calcium phosphate dihydrate [138]. Various types of carbohydrate coatings can be incorporated to adsorb onto the core to form a water-like surrounding that determines the conformational stability of encapsulated drugs [137]. Examples of carbohydrates used are lactose, trehalose, sucrose, cellobiose and pyridoxal phosphate [138]. High encapsulation efficiency, minimal drug leakage, chemical stability and pulsatile drug release profiles brought aquasomes into view to address the challenges in the current available line-up of nanovesicle formulations [137, 138]. Aquasomes are specifically useful for the transport of chemically labile drugs due to their inert properties to protect drugs from degradation, i.e. oxidation or hydrolysis, reported with niosomes and transfersomes [138]. Their intrinsic advantages lead to the development of marketed formulations with substantial use in the medical field, e.g. Optaflu (cell-based seasonal influenza vaccine) and Aranesp (darbepoetin for treatment of anaemia) [138].

Cephalothin belongs to the class of cephalosporins, which are commonly used to treat SSTIs. Cephalothin-loaded aquasomes exhibited a ZOI of 38 ± 3 mm against *S. aureus* compared to 29 ± 5 mm with cephalothin alone [139]. The authors confirmed the sustained release profile of cephalothin-loaded aquasomes with 60.72% cephalothin release over 10 h which is likely to leverage the time-dependent antibacterial effect of cephalothin. It would be worth to proceed with further investigation of the in vivo antibacterial effect with topical formulations of cephalothin-loaded aquasomes, owing to their exceptional characteristics.

### Lipid nanoparticles

Solid lipid nanoparticles (SLNs) and nanostructured lipid carriers (NLCs) are the two main types of lipid nanoparticles. Typically, lipid nanoparticles are used to carry poorly water-soluble agents by loading them in a lipid matrix. SLNs consist of only solid lipids which comprise of mainly longer chain structures [110, 140]. Nonetheless, the crystallisation of SLNs occurs at room temperature which is likely to cause drug expulsion [140]. Crystallised structures of SLNs also restrict the encapsulation efficiency because of high rigidity. NLCs are known as the newer generation of lipid nanoparticles developed to overcome the shortcomings of SLNs associated with low drug loading capacity [110, 140]. NLCs contain a mixture of solid and liquid lipids stabilised by surfactants intercalated in the emulsifier layers to form a spherical configuration. The presence of liquid lipids gives rise to the advantages of enhanced flexibility, fluidity, increased surface area of contact and more capacity for drug entrapment attributed to their more disordered structure [141–145]. NLCs represent a promising alternative to produce topical formulations of antimicrobial agents (Table 4).

**Table 4:** Summary of studies on in vitro cytotoxicity and antibacterial activity of lipid nanoparticles

Type of NP	Active ingredient	Hydrodynamic diameter (nm)	Zeta potential (mV)	EE (%)	Bacteria	In vitro efficacy findings	Cytotoxicity findings	Reference
SLN-based gel	Linezolid	206.3 ± 0.17 ^a^	-24.4 ± 4.67	80.90 ± 0.45	*S. aureus* ^*^	• ZOI for *S. aureus* was 5.03 ± 0.15 cm • Gel base did not exhibit any ZOI	-	[146]
NLC-based gel	Mupirocin	150.25 ± 1.52 ^b^	−15	88.02 ± 1.21	*S. aureus* ^*^	• ZOI: standard (3.4 ± 0.20 mm) > NLC based gel (3.3 ± 0.10 mm) > marketed formulation (3.1 ± 0.18 mm) > NLC formulation (2.9 ± 0.12 mm)	-	[145]
NLC	Meropenem hydrochloride	126.5 ± 0.9 ^b^	+ 0.967	79.1 ± 2.3	*S. aureus* (MTCC 737)	• ZOI of 22 mm with MpM-NLC gel and 18 mm with MpM plain gel	-	[144]
NLC	Quercetin	160 to 185 ^a^	> −40	> 99%	*S. aureus* ^*^	• QR loading reduced MIC compared to empty NPO-NLCs: QR-SF-NLC (from 6.25 to 0.78 mg/mL), QR-OV-NLCs (from 3.13 to 0.78 mg/ mL), QR-CC-NLC (from 6.25 to 0.16 mg/mL)	• Reference used: ISO 10993-5:2009 • QR-NPO-NLCs increased HaCaT cell viability compared to free QR and empty NPO-NLCs	[143]
Cationic NLC	Oxacillin	177.00 ± 9.55 ^b^	18.70 ± 0.82	76.8 ± 7.0	*S. aureus* (ATCC 6538), MRSA (ATCC 33591 and KM1)	• MBC of oxacillin alone against non-resistant *S. aureus* was 0.488–0.976 µg/mL, SME in NLC was 62.5 µg/mL • Combined NLC and oxacillin reduced SME MBC by 16-fold but not MBC of oxacillin • Oxacillin + NLC reduced MBC of oxacillin for MRSA from 250 to 62.5 µg/mL • Oxacillin + NLC reduced MBC of oxacillin for clinical MRSA strain (KM1) from 62.5–125 to 7.812 µg/mL • MBC of SME in NLC decreased two-fold after oxacillin entrapment	-	[141]

Abbreviations: Coconut oil, CC; Encapsulation efficiency, EE; Meropenem, MpM; Methicillin-resistant *Staphylococcus aureus*, MRSA; Minimum bactericidal concentration, MBC; Minimum inhibitory concentration, MIC; Nanoemulsion, NE; Nanoemulgel, NEG; Nanoparticle, NP; Nanostructured lipid carrier, NLC; Natural plant oil, NPO; Olive oil, OV; Quercetin, QR; Solid lipid nanoparticle, SLN; *Staphylococcus aureus*, *S. aureus*; Sunflower oil, SF; Zone of inhibition, ZOI

^a^ Size measured using dynamic light scattering (DLS)

^b^ Size measured using transmission electron microscopy (TEM)

^*^ Isolate used unspecified

#### Solid lipid nanoparticles

Linezolid is a synthetic antibiotic known as oxazolidinone that provides antibacterial coverage against Gram-positive bacteria, notably as the choice of drug to combat MRSA. One study declared a ZOI of 5.03 ± 0.15 cm with linezolid-loaded SLN-based gel for *S. aureus* compared to zero ZOI with placebo gel base, indicating the linezolid-loaded SLN was solely responsible for the antibacterial effect [146]. To simulate superficial SSTIs, a *S. aureus* count of 4.533 ± 0.243 (log_10_ CFU) was determined using a tape-stripping rat skin infection model treated topically with linezolid-loaded SLN-based gel, which was significantly lower than negative control group, 6.915 ± 0.129 (log_10_ CFU) (*P* < 0.0001) [146]. As linezolid exhibits time-dependent killing, SLNs can act as a reservoir attributed to the lipid matrix for a controlled release profile which can potentiate the antibacterial activity by prolonging the duration of on-site antibiotic concentration above MIC. This was proven by the sustained linezolid release pattern of 92.06 ± 4.68% and 85.43% after 24 h from SLN and SLN-based gel, respectively [146]. Results suggest that SLNs can be potentially formulated into topical gel for dermal drug delivery as a treatment strategy of uncomplicated SSTIs. However, the authors did not conduct antibacterial tests with free linezolid, thus lacking comparison data in acclaiming whether incorporation of linezolid into SLNs truly enhances the antibacterial effect.

#### Nanostructured lipid carriers

Shinde et al. reported greater ZOI of *S. aureus* treated with a mupirocin-loaded NLC based gel (3.3 mm) as compared to a marketed mupirocin cream equivalent to 20 mg (3.1 mm) [145]. Another study by Rajpoot et al. also found greater ZOI against *S. aureus* (MTCC 737) treated with meropenem-loaded NLC gel (22 mm) versus meropenem alone (18 mm) that supported the previous findings of improved antibacterial efficacy attributed to their extended-release properties [144]. Both mupirocin, a macrolide and meropenem, a beta-lactam possess time-dependent antibacterial activity (T > MIC). Thus, extended-release formulations can maintain drug concentrations above the MIC for longer periods which would be more effective than immediate-release formulation. The controlled release profile of NLCs enhances drug retention by maintaining a sustained concentration gradient across the skin, highlighting their potential as effective nanocarriers for topical SSTI treatment.

Previously, one study by de Barros et al. demonstrated antimicrobial activity of unloaded natural plant-oil-based NLCs (NPO-NLCs) against *S. aureus* [142]. NLCs can be utilised for topical dermal drug delivery or even as antibacterial agents themselves depending on the inherent antibacterial activity of surface-active agents added. In 2022, the authors further investigated quercetin, a flavonoid extracted from plant sources encapsulated into NPO-NLCs [143]. Their study obtained a MIC reduction with quercetin-loaded NPO-NLCs compared to empty NPO-NLCs [143]. These findings propose that the incorporation of bioactive compounds into NLCs can bring additive antibacterial effects. Similarly, Alalaiwe et al. revealed the additive effect of oxacillin-loaded NLCs which reduced the MBC of SME by 16-fold (62.5 µg/mL to 0.488–0.976 µg/mL) and 2-fold against *S. aureus* (ATCC 6538) and MRSA (ATCC 33591 and clinical isolate), respectively [141]. There was no fractional inhibitory concentration (FIC) assay conducted in those studies thus no definitive evidence of synergism can be drawn from the data presented by the authors. It would be worth consideration for the authors to incorporate checkerboard or FIC assays to determine the synergistic activity of active compounds and the developed nanoparticle system in the future.

### Polymeric nanoparticles

Polymeric nanoparticles are composed of either naturally occurring polymers or synthetic polymers. Natural polymeric nanoparticles are particularly useful in formulating topical skin delivery systems for hydrophilic agents, due to their inherent tendency to form hydrogels via electrostatic interactions. Examples of natural polymers for topical applications include chitosan, gelatin, albumin, starch and alginate [147, 148]. Synthetic polymeric nanoparticles offer greater reproducibility, less variability, and higher purity, owing to standardized manufacturing processes, compared to natural polymeric nanoparticles. Common synthetic polymers used to produce nanoparticles can be classified as biodegradable (e.g. polyesters) or non-biodegradable (e.g. polyacrylates), which can be readily adjusted to carry either hydrophilic or lipophilic therapeutic cargo [149]. Use of block copolymers as building blocks imparts desirable properties to the resulting polymeric nanoparticles with optimisations for targeted topical drug delivery such as polymersomes, nanocapsules, nanogels and nanospheres, exhibiting improved drug solubility, skin penetration and formulation stability [149–152]. Polymeric nanoparticles have been extensively employed in both cosmetic and pharmaceutical fields for safeguarding the drug potency from biological interactions to preventing physical and chemical instability (Table 5) [148, 149, 152–155].

**Table 5:** Summary of studies on in vitro cytotoxicity and antibacterial activity of polymeric nanoparticles

Type of NP	Active ingredient	Hydrodynamic diameter (nm)	Zeta potential (mV)	EE (%)	Bacteria	In vitro efficacy findings	Cytotoxicity findings	Reference
Poly(ϵ-caprolactone) nanocapsule	Chlorhexidine	-	>+30	60	*S. aureus* (clinical isolate)	• Both MIC of 0.15% CHX base nanocapsules and 0.15% CHX digluconate for *S. epidermidis* and *S. aureus* were 1.2 µg/mL	-	[151]
Sustained-release-polycaprolactone NP	Quercetin & copper–quercetin (Cu–Q) complex	Q-PCL-NPs: 324.5 ^c^ Cu-Q-PCL-NPs: 994.9 ^c^	-	93.94	*S. aureus* (clinical isolate: MR.2209243)	• ZOI of Cu-Q-PCL-NPs against S. aureus (20 to 50 mm) was greater than Q-PCL-NPs and empty PCL-NPs	-	[156]
Chitosan nanoparticles	Chlorhexidine digluconate (CHX)	241.7 ± 22.2 ^a^	35.87 ± 0.6	84.2 ± 1.9	*S. aureus* (ATCC 29213)	• MIC of gel formulation Chi-MN-CHX^NP^-TE, Chi-MN-CHX-TE, Chi-CHX-TE and Chi-MN-TE against *S. aureus* were 0.016, 0.20, 0.49 and 3.91 µg/mL, respectively	Gel formulation Chi-MN-CHX^NP^-TE showed the highest cell viability (> 95%) to L929 mouse fibroblast cells	[154]
Chitosan-alginate nanoparticles	Oregano Oil	350 ^b^	-	-^+^	*S. aureus* (ATCC 29213), MRSA (NBIMCC 8327)	• MIC of OrO-NP-HG was 0.0625% against both MRSA and *S. aureus* • MIC of CN-HG was 0.25 mg/mL both MRSA and *S. aureus*, while MICs of CIP-HG were 0.25 mg/mL against *S. aureus* and 0.5 mg/mL against MRSA, respectively • 4-fold reduction in MIC was observed with 0.5× MIC OrO-NP-HG added to CIP-HG against MSSA • 8-fold reduction in MIC was observed with 0.5× MIC OrO-NP-HG added to CIP-HG against MRSA • 16-fold reduction in MIC was observed with 0.25× MIC OrO-NP-HG added to CN-HG against MSSA and MRSA	-	[155]
Dialdehyde starch NP	Vancomycin	76 ^a, c^	-15	58.1	*S. aureus* (ATCC 25923)	• Inhibition zones were evaluated using Kirby-Bauer (KB) test • ZOI of SF, SF/VAN and SF/DASNP/VAN were zero, 14 mm and 12 mm, respectively	Cell viability of 69% with SF/DASNP/VAN to L929 fibroblast cells	[159]
Nanohydrogel	Cephalexin	178 ± 6.31 ^b^	7.23 ± 1.05	58 ± 5.26	*S. aureus* ^*^	• MICs of cephalexin-loaded NPs and untreated cephalexin were 1 µg/mL and 4 µg/mL (*P* < 0.05), respectively	-	[161]
Hyaluronan-cholesterol nanohydrogels	Gentamicin and levofloxacin	NH/LVF: 350 ^b^ NH/GM: 250 ^b^	-	NH/LVF: 11.4 ± 3.1 NH/GM: 40.0 ± 1.0	*S. aureus* (NCTC 12973)	• Same MIC and MBC between free drug and drug-loaded NHs • NH/GM: both MIC and MBC were 15.6 µg/mL • NH/LVF: MIC and MBC were 11.7 and 15.6 ± 5 µg/mL	Both drug-loaded NHs were not toxic to HaCaT after 48 h of treatment	[160]
Chitosan Nanogel	Mupirocin	341.920 ± 15.201 ^a^	10.340 ± 0.607 to 24.300 ± 0.775	97.00 ± 3.12	*S. aureus* (ATCC 29213)	• In disk diffusion test, mean ZOI with MPR was 32.67 ± 0.58 mm • In agar well diffusion test, mean ZOI with MPR was 33.33 ± 0.58 mm • MPR-loaded nanogels had no significant difference compared to MPR in both tests	MPR-loaded nanogels exhibited CCD-1070Sk (ATCC^®^ CRL-2091) viability above 90% over 24, 48 and 72 h of treatment	[162]

Abbreviations: Chitosan, Chi; Chlorhexidine, CHX; Chlorhexidine gluconate and miconazole nitrate-loaded chitosan nanoparticles added to Tris-EDTA in chitosan gel, Chi-MN-CHX^NP^-TE; Chlorhexidine, CHX; Ciprofloxacin, CIP; Copper, Cu; Dialdehyde starch nanoparticles, DASNP; 2,2-diphenyl-1-picrylhydrazyl, DPPH; Encapsulation efficiency, EE; Gentamicin, CN; Gentamicin, GM; Hydrogel, HG; Levofloxacin, LVF; Methicillin-sensitive *Staphylococcus aureus*, MSSA; Methicillin-resistant *Staphylococcus aureus*, MRSA; Miconazole nitrate, MN; Minimum bactericidal concentration, MBC; Minimum inhibitory concentration, MIC; Mupirocin, MPR; Nanohydrogel, NH; Nanoparticle, NP; Oregano oil, OrO; Phosphate buffered saline, PBS; Polycaprolactone, PCL; Quercetin, Q; Silk fibroin, SF; *Staphylococcus aureus*, *S. aureus*; *Staphylococcus epidermidis*, *S. epidermidis*; Tris-EDTA, TE; Vancomycin, VAN; Zone of inhibition, ZOI

^a^ Size measured using dynamic light scattering (DLS)

^b^ Size measured using transmission electron microscopy (TEM)

^c^ Size measured using scanning electron microscopy (SEM)

^*^ Isolate used unspecified

^+^ Data not reported

#### Nanospheres

A study by Ramzan et al. (2023) tested copper-quercetin complex-loaded polycaprolactone nanoparticles (Cu-Q-PCL-NPs) against common SSTI pathogens that cause impetigo. By combining both metallic copper and a phytochemical such as quercetin in the form of a metal-complex and encapsulating it within a polycaprolactone nanoparticle, the authors expected to obtain greater antibacterial activity [156]. Findings from the study showed significant increments in the ZOI against clinical isolates of *P. aeruginosa* (MR.1047313) and *S. aureus* (MR.2209243) in the Cu-Q-PCL-NP treatment group (50 mm) compared with quercetin (20 mm) [156]. It was proposed that the alteration in functional groups of quercetin involved in the formation of Cu-Q complex potentiated interaction with the bacterial surface to initiate cell death [156]. Despite the dominance of polymeric nanoparticles, further innovative strategies such as constituent attachment and bioactive compound complexation are typically necessary to achieve uniform penetration and effective drug potency [157].

Chitosan is a natural polymer, widely known for its inherent antibacterial activity due to its polycationic nature. Turkmen et al. found that when chlorhexidine gluconate and miconazole nitrate-loaded chitosan nanoparticles were added to Tris-EDTA in chitosan gel (Chi-MN-CHX^NP^-TE), the MIC against *S. aureus* (ATCC 29213) was reported to be 0.016 µg/mL, compared to 0.20 µg/mL of the formulation without nanoparticle (Chi-MN-CHX-TE) [154]. Zaharieva et al. also reported MIC reduction by 8-fold for ciprofloxacin (CIP), a fluoroquinolone, and 16-fold for gentamicin (CN), an aminoglycoside, when used in combination with oregano oil-loaded chitosan alginate nanoparticles hydrogel (OrO-NP-HG) against MRSA (NBIMCC 8327) [155]. The authors further conducted a checkerboard assay which data can be analysed based on the sum of both FIC values (Σ*FIC*): synergy (Σ*FIC* ≤ 0.5), indifference (0.5 *<* Σ*FIC* ≤ 4), additive (0.5 *<* Σ*FIC* ≤ 1) and antagonism (Σ*FIC* > 4). OrO-NP-HG + CN-HG displayed a Σ*FIC* of 0.312 indicating synergism against MRSA, while OrO-NP-HG + CIP-HG showed 0.625, portraying additive antibacterial effect against MRSA [155]. Findings suggest that incorporating antimicrobial agents into chitosan nanoparticles helps with controlled drug release as well as synergistic or additive antibacterial effects which is essential to reduce the unnecessary antibiotic exposure and associated risk of toxicity for the treatment of SSTIs.

Starch, a natural polysaccharide, has been widely employed in targeted and sustained drug delivery systems due to its stability, biocompatibility, and versatile interaction capabilities with other molecules [158]. A starch-based nanoparticulate system (StNC) demonstrated higher skin retention and permeation profiles compared to free drug solution through in vitro studies using newborn pig skin, indicating its potential for topical drug delivery applications [62]. A silk fibroin (SF) dressing incorporating vancomycin-loaded dialdehyde starch nanoparticles (SF/DASNP/VAN) exhibited a slightly smaller ZOI of 12 mm against *S. aureus* (ATCC 25923) compared to the dressing loaded with free vancomycin (SF/VAN), which showed a ZOI of 14 mm [159]. This observation was attributed to the controlled drug release from the SF/DASNP/VAN formulation over a 48 h period, resulting from the interaction between the aldehyde groups of DAS and the amine groups of vancomycin, which slows the drug release compared to SF/VAN [159]. The elegant design developed by the authors showcases the versatility of natural polymers such as starch complementing the chemical structure of vancomycin to improve drug delivery. By taking advantage of this niche aspect, the authors have shown starch nanoparticles hold excellent promise for the delivery of vancomycin to treat SSTIs.

A recent study by Thamilselvan et al. investigated the in vivo topical application of dual drug-loaded polyvinyl alcohol (PVA) based hydrogels containing an efflux pump inhibitor, 5-Nitro-2-(3-phenylpropoxy) pyridine (5-NPPP) loaded polymeric nanoparticles and CIP in combination against *S. aureus* by using an in vivo mice skin infection model [153]. Dual-drug-loaded hydrogels demonstrate enhanced efficacy in clearing infections by inhibiting bacterial efflux mechanisms, thereby facilitating higher intracellular CIP accumulation [153]. As a fluoroquinolone antibiotic, CIP exhibits concentration-dependent antibacterial activity, leading to a more potent bactericidal effect with a higher Cmax. Findings propose that dual drug combination is an effective strategy to combat resistant strains by restoring bacterial susceptibility to CIP. However, the efficacy of this combinatorial treatment may be compromised by mismatched drug release kinetics, as 5-NPPP acts by blocking CIP resistance mechanisms rather than exerting direct antibacterial activity. The optimised 5-NPPP loaded polymeric nanoparticles demonstrated a linear sustained drug release for 2 days compared to pure 5-NPPP solution with cumulative maximum 100% release after 1 day [153].

#### Nanogels

Nanogels fall under the large umbrella of polymeric nanoparticles and given their own subset owing to their unique characteristics which sets them apart. Nanogels are soft, thermosensitive, crosslinked three-dimensional structures with swelling properties which plays a key role in controlling the rate of drug release [160, 161]. The gelling system allows them to be highly responsive to environmental stimuli such as pH, temperature, enzymatic action and water content thus higher flexibility in designing smart drug delivery systems [160, 161]. Nanogels also exhibit increased bioadhesion, enhanced formulation retention and drug deposition in the skin compared to conventional hydrogels which can be a promising approach to develop novel topical nano therapy [162].

Salatin et al. developed a cephalexin nanohydrogel (NH) for the goal of treating impetigo. On day 6 of topical application onto the in vivo *S. aureus*-infected rat skin models, the authors documented the bacterial counts of 2.51 ± 2.60% with cephalexin NH and 28.55 ± 3.01% with cephalexin hydrogel relative to that of the untreated control, respectively [161]. Presence of nanoparticles in the hydrogel system favours sustained release kinetics as well as better adsorption to the bacterial surface due to the increased surface area-to-volume ratio, thus targeting the metabolic pathways with high intracellular antibiotic concentrations. This characteristic is particularly useful for drugs with rapid clearance as the diffusion of loaded drug into the hydrogel network delays the release until reaching the site of infection within the skin layers.

In the context of SSTIs, intracellular pathogens can complicate antibiotic treatment by serving as a barrier for effective drug delivery. As a case example, intracellular *S. aureus* has been found to typically reside in the lysosomes of cells, which causes difficulty in eliminating the pathogen from the host [160]. Montanari et al. reported enhanced intracellular antibacterial activity of hyaluronan-cholesterol nanohydrogels loaded with levofloxacin (NH/LVF) compared to LVF (*P* < 0.05) secondary to accumulation of NH/LVF in the lysosomes of human keratinocytes (HaCaT) as evidenced by a fluorescence uptake study, compared to the buildup of cytosolic free LVF against intracellular *S. aureus* (NCTC 12973) [160]. Incorporation of antibiotics into NHs can potentially alter the final destination of drugs depending on the intracellular location of pathogens, e.g. intracellular *S. aureus* in human keratinocytes to improve SSTI eradication.

## Clinical trials and patent trends

Current patent trends can predict the direction of future research and development in the field of interest. Translational aspects of academic research can also be drawn in a clearer light by inferring from current patent trends. Data on the fabrication of the nanoparticle-based topical skin therapies for bacterial SSTIs up to 2025 was obtained through a patent search. This review involved an in-depth critical screening of patents on the international database lens.org that interconnects patent offices of the US and World Intellectual Property Organization (WIPO) having 193 member states around the globe. The search queries applied were (i) “nanoparticle” in [Title/Abstract/Claims] and “skin and soft tissue infection” in [Title/Abstract/Claims] and (ii) “nanoparticle” in [Title/Abstract/Claims] and “topical” in [Title/Abstract/Claims]. There was a total of 35 patent records procured as a result. Only 7 records were extracted based on the abstract description and patent claims relevant to the topical nanoformulations for the treatment of bacterial SSTIs (Table 6).

**Table 6:** Patents pending up to 2025 in relation to topical dermal drug delivery nanosystems for the treatment of bacterial SSTIs

No.	Patent no.	Year	Patent title	Inventor (s)	Topical formulation	Reference
1	WO 2015/080670 A1	2015	Novel ultrashort hydrophobic peptides that self-assemble into nanofibrous hydrogels and their uses	Hauser and Loo	Nanohydrogels	[164]
2	WO 2015/110,957 A2	2015	Hybridosomes, compositions comprising the same, processes for their production and uses thereof	De Beer	Liposomes, cerasomes, sphingosomes, niosomes, polymerosomes, lipid-based nanoparticles	[166]
3	WO 2018/226,479 A1	2018	Compositions and methods for treating wounds	Noel et al.	Silver nanoparticles	[168]
4	US 2020/0129564 A1	2020	Compositions and methods for treating wounds	Noel et al.	Silver nanoparticles	[169]
5	WO 2022/077060 A1	2022	Antimicrobial compositions and methods of use	Thorn et al.	Nanostructured liquid crystal carriers	[171]
6	US 2023/0390316 A1	2023	Antimicrobial compositions and methods of use	Thorn et al.	Nanostructured liquid crystal carriers	[172]
7	WO 2023/248,196 A1	2023	Peptide-ionic liquid conjugate for the prevention and/or treatment of skin disorders	De Carvalho et al.	Solid lipid nanoparticles, nanostructured lipid carriers, liposomes, polymeric nanoparticles, nanohydrogels	[173]

To date, there is no granted patent but only patent applications from 2008 to 2025. Limited number of patent applications throughout the years may be due to a lack of promising clinical trials with topical skin drug delivery nanosystems in treating bacterial SSTIs which impede the confidence in transitioning to filing a patent. Using the database, clinicaltrials.gov, the search using the same search strategy yielded neither ongoing nor completed clinical trials with nanoparticle-based topical skin treatments for bacterial SSTIs. From examining the previous literature, this review was able to identify only 1 double-blinded clinical trial in 1999 testing topical liposomal clofazimine (CLO) on eight leprosy patients [163]. Results of the trial corroborated the expectation of researchers about prolonged skin residence time with liposomal CLO gel over plain CLO gel, reducing the duration of therapy for leprosy [163]. Inadequate efficacy and safety data of the topical nanoformulations from pre-clinical studies could be the main reason of minimal clinical trials, and the field stands in need of further laboratory investigation. Subsequently, the following section discusses the pending applied patents extracted from the patent database.

Two patent applications were found in the year of 2015. Patent WO 2015/080670 A1 presented the invention of a non-toxic and highly rigid nanofibrous hydrogel using self-assembled hydrophobic peptides or peptidomimetics with “biofabrication” techniques [164]. Nanofibrous hydrogels are known to be more advanced by their capability to mimic the in vivo biological variability such as extracellular matrices and react to the dynamic cellular changes in terms of biochemical signalling and physiological adaptation compared to conventional hydrogels [165]. The invention was said to be suitable for pharmaceutical delivery of antimicrobial agents in the form of topical gels or creams to treat infections [164]. Patent WO 2015/110,957 A2 published the invention of hybridosomes by synergising the pros of synthetic nanocarriers and naturally occurring extracellular vesicles to introduce a novel engineered nano-particular drug delivery system [166]. The inventor proposed that hybridosomes can be potential therapeutic carriers which offer multiple routes of administration including topical skin therapy to be highlighted.

From 2015 onwards, the patent trend has been revolving around discovery of alternatives and addressing the root causes of treatment failure such as biofilm growth found in most bacterial SSTIs. It is believed and recognised that the emergence of antibiotic resistance over the years drastically restricts the availability of treatments options despite previous efforts in finding novel agents [167]. Patent applications WO 2018/226,479 A1 and US 2020/0129564 A1 established an invention of topical formulations containing AgNPs including liquid, gel, paste, semi-solid and solid dressing or bandage to treat superficial and soft tissue infections which are usually associated with presence of wounds. Noteworthy characteristics of the invention included a viscosity greater than 500 centipoise (cP) and their antibacterial activity against Gram-negative or Gram-positive bacteria [168, 169]. Viscosity is a crucial factor to determine the tendency of topical formulation to retain against skin surface after application which in turn influences the therapeutic efficacy [161]. Patents application WO 2022/077060 A1 and US 2023/0390316 A1 showcase the development of a nanostructured liquid crystal carrier containing aminoglycosides which are cationic antibiotics such as tobramycin, gentamicin, and amikacin that introduced them as topical skin therapies to treat Gram-negative infection. The invention proved to exhibit superior antibacterial activity in addition to anti-biofilm properties compared to antibiotics alone regarding their performance in eradicating infections associated with biofilms which is always a vital drawback of current treatment options [167, 170–172].

The latest patent application WO 2023/248,196 A1 in 2023 was related to topical nanoformulations encapsulating peptide-ionic liquid (PIL) conjugate indicated for skin disorders including bacterial skin infections. The present invention was formulated by chemical modification involving direct coupling of alkylimidazolium-based IL to CPs which conferred antimicrobial properties of the conjugates. Ionic liquid acts as a CPE while CPs promote skin healing [173]. It was claimed that topical nano delivery of peptide-ionic liquid conjugates can effectively treat SSTIs with enhanced dermal permeation and rapid skin regeneration [174]. However, more studies are needed to investigate the outcomes of incorporating PIL into nanoformulations in the context of toxicity profile, resistance to proteolytic degradation and retention rate at the infected areas of skin after topical application.

## Conclusion and future prospects

SSTIs are rising to be a global problem especially with growing antibiotic resistance amongst a community in an urgent demand for a solution to this plight. Development of novel drug delivery systems seems to be a viable solution by maximising the usage of currently accessible antibacterial agents to mitigate the likelihood of resistance. In favour of local therapeutic effect for SSTIs, topical dermal therapies took precedence over any other routes of drug delivery to avoid redundant systemic exposure to antibiotics. Studies presented in the current review have highlighted the potential role of nanosystems in topical nanoformulations that enhanced treatment efficacy, safety, and efficiency while more importantly showcased interesting aspects and mechanisms in overcoming barriers for treating SSTIs.

Topical nanoformulations were designed to address the constraints of conventional formulations in the context of effective dermal drug delivery for the treatment of SSTIs. It was evident that fabrication of topical nanoformulations demonstrated notable antibacterial activity against the common SSTI-causing Gram-positive bacteria, including MRSA based on MIC, MBC, and CFU, which makes them a considerable alternative management for SSTIs. Several studies have claimed synergistic effects from combinations of antimicrobial agents; however, such assertions are not sufficiently valid without supporting data from checkerboard or FIC assays. To standardise antimicrobial testing protocols, researchers are strongly encouraged to adhere to the Clinical and Laboratory Standards Institute (CLSI) guidelines. This compliance facilitates better assimilation of study outcomes and enhances their translation into clinical practice by allowing better comparability of data between separate studies.

**Fig. 6:**
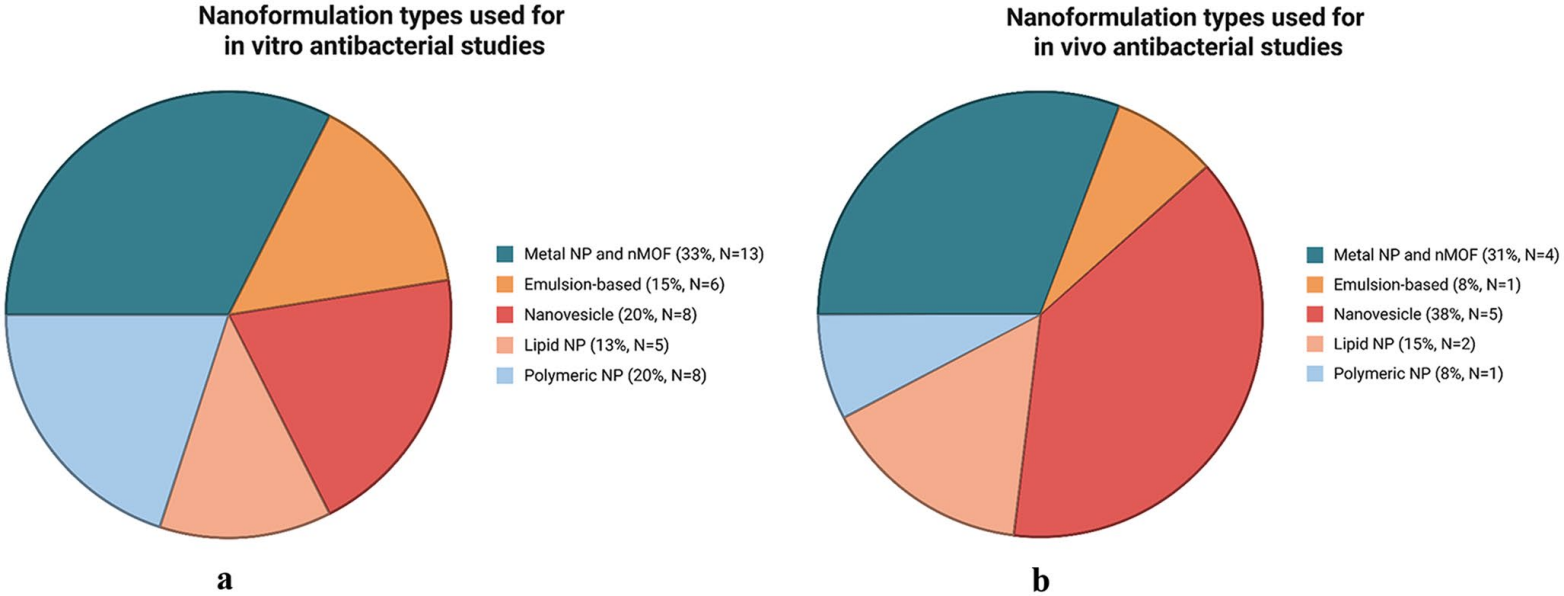
**a** Topical nanoformulations used for in vitro antibacterial testing. **b** Topical nanoformulations used for in vivo antibacterial testing

As shown in Fig. 6a, this review found that the majority of in vitro antibacterial studies focused on topical metallic NPs and nMOFs, while lipid NPs were the least represented based on the latest literature search (up to April 2025). Despite encompassing the majority of in vitro efficacy studies, the absence of corresponding safety profiles impedes the progression of inorganic metallic nanoparticles (NPs) to in vivo evaluation. In contrast, nanovesicles have emerged as the most frequently studied formulation using in vivo models (Fig. 6b), likely due to their comparatively robust cytotoxicity profiles. In vitro cytotoxicity assessment stands out as a key preclinical translational hurdle, contributing to the observed disproportion and translational gap in developing topical nanoformulations for SSTIs. This trend may also be driven by the evolutionary advancement from conventional liposomes to the newer generation vesicular nanoparticulate systems, which have overcome the limitations of earlier designs. Ongoing innovation in nanovesicle technology has further expanded their potential in biomedical and pharmaceutical applications, garnering increased research interest relative to other nanoformulations, particularly in SSTIs as demonstrated in this review.

**Fig. 7:**
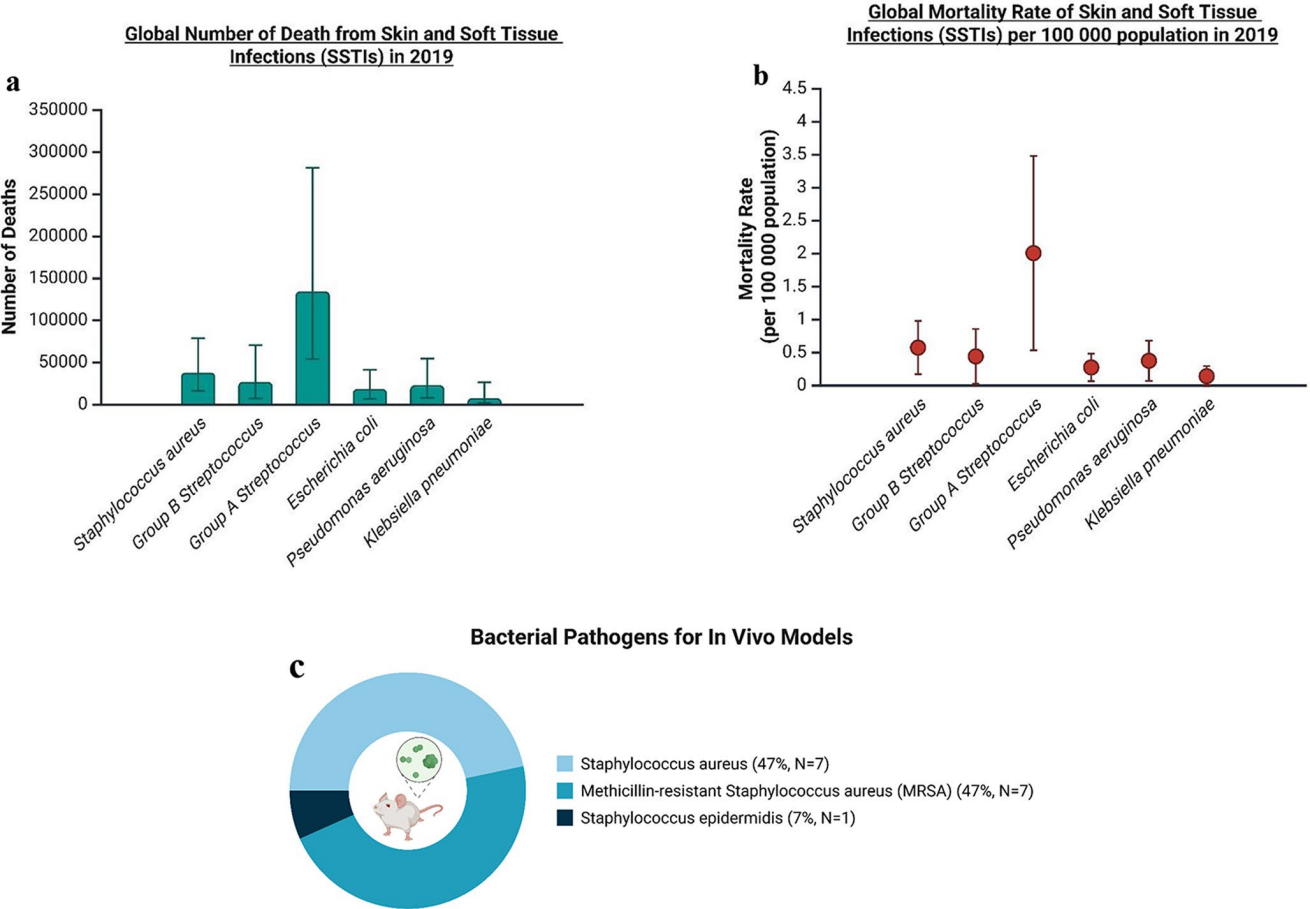
**a** Global number of deaths from SSTIs in 2019 [175]. Data from *The Lancet*, 400, Ikuta, K. S. et al., Global mortality associated with 33 bacterial pathogens in 2019: a systematic analysis for the Global Burden of Disease Study 2019, 2221–2248, Copyright Elsevier (2025). **b** Global mortality rate from SSTIs in 2019 [175]. Data from *The Lancet*, 400, Ikuta, K. S. et al., Global mortality associated with 33 bacterial pathogens in 2019: a systematic analysis for the Global Burden of Disease Study 2019, 2221–2248, Copyright Elsevier (2025). **c** Bacterial pathogen used for in vivo skin infection models [74, 83, 97, 98, 104, 111, 115, 116, 119, 141, 146, 153]

In vivo antibacterial evaluations have predominantly focused on Gram-positive bacteria, with limited investigation of Gram-negative pathogens (Table 7), despite most in vitro studies covered for both Gram-positive and Gram-negative bacteria. Disparities between the bacterial populations used for in vivo models compared to the in vitro models can be explained by a higher prevalence of Gram-positive pathogens in causing uncomplicated SSTIs, leading to greater clinical significance of further translation. According to a systematic analysis from the GBD 2019, published in *The Lancet*, the majority of global SSTI-related deaths were attributed to Gram-positive bacteria (Fig. 7a) [175]. Although Group A *Streptococcus* was identified as the most prevalent cause of SSTI-related mortality, the estimates for both the global number of deaths and the mortality rate per 100,000 population were accompanied by relatively wide 95% uncertainty intervals compared to other bacterial pathogens, suggesting lower precision in these figures (Fig. 7b) [175].

**Table 7:** Summary of studies on in vivo antibacterial activity of topical application of nanoparticle-based therapies

Type of NP	Active ingredient	Hydrodynamic diameter (nm)	Zeta potential (mV)	EE (%)	Bacteria	Animal model	In vivo efficacy findings	Reference
AgNP	-	10 to 30 ^b^	-	-	MRSA (ATCC 14458)	Male mice	• Bacteria counted for topically treated with control, AgNPs, allicin and their combination were 3.77 × 10^10^, 8.0 × 10^7^, 4.3 × 10^6^, and 0 CFU/mL, respectively	[74]
ZnONP	-	10 to 50 nm ^b^	-	-	*S. aureus* (ATCC 25923 and ATCC 29213)	BALB/c mice	• ZnONPs exhibited almost 40% reduction in bacterial burden when compared to untreated control (*P* < 0.005) • Histopathological result: Thinning of epidermal layer and large number of inflammatory cells observed in *S. aureus* infected skin; ZnONPs treated skin showed less damage of epidermal layer and healing to recover normal intact structure	[83]
Nanofiber	Vancomycin	201 ± 67 ^c^	-	63 ± 10	MRSA (clinical isolates)	Rat skin abrasion model	• Nanofibers significantly decreased bacterial counts compared to free vancomycin (0.05 > *P* ≥ 0.01) • No mortalities in nanofiber treated groups	[97]
DNC	-	38.9 ± 1.0 ^a^	−31.6 to − 49.4	-	MRSA (clinical isolate)	C57BL/6 mice	• Topical application of DNC (5 mg/mL) significantly decreased CFU of MRSA and reduced skin inflammation in all 3 skin infection models similar to those treated with gentamicin sulphate	[98]
Nanoemulsion and liposome	Soyaethyl morpholinium ethosulfate (SME)	LP: 75.0 ± 7.8 ^a^ NE: 214.4 ± 1.6 ^a^	LP: 38.2 ± 4.2 NE: 54.1 ± 0.1	-	MRSA^*^, S. aureus^*^, S. epidermidis^*^	BALB/c mice	• Topical application of both nanosystems significantly decreased the wound’s infection while edema was still noticeable with liposome treatment • LP-treated infected skin had mild hyperkeratosis and chronic inflammatory cell infiltration while NE-treated infected skin had no abnormalities • LP-treated skin had decreased CFU by > 200-fold and further reduction in NE-treated skin	[104]
Ethosomes	Linezolid	186 to 346 ^a^	-	42.95 to 69.59	*S. aureus* ^*^	Diabetic Sprague Dawley rats	• Histopathological evaluation of streptozotocin induced diabetic rats showed no bacterial growth, re-grew hair and full recovery from the infection after 14 days with LZD-loaded ethosomes treatment compared to control which exhibited significant necrosis of skin tissue and formation of crust	[115]
Terpesome	Levocetirizine dihydrochloride	243.3 ± 4.60 ^b^	23.20 ± 0.55	69.64 ± 0.14	MRSA (USA300)	BALB/C female mice	• Bacterial count in TPs-gel topically treated group was 3.14 (log10 CFU) (*P* < 0.01), while levocetirizine dihydrochloride gel (LVC-gel) was 1.62 (log10 CFU) (*P* < 0.01) lower than the negative control and vehicle control groups	[111]
Novasome	Luteolin	383.4 ± 19.7 ^b^	-7.9 ± 2.57	79.8 ± 1.9	MRSA (clinical isolates: MS3, MS15, MS16, MS17, MS23 and MS43)	BALB/C female mice	• Noticeable healing of lesions and significant reduction in microbial load in groups treated with LUT-loaded novasomes and FA	[119]
NT chitosan hydrogel	Cephalexin	< 200 ^c^	-17.6	35.06 ± 2.96 to 71.11 ± 3.52	*S. aureus* ^*^	male Wistar rats	• On day 3 of topical treatment with NT hydrogel, plain hydrogel and chitosan-treated rat groups showed a bacterial count of 40.67 ± 10.26, 50.94 ± 8.07 and 90.27 ± 2.00% compared to the untreated control, respectively • Complete wound healing with the use of topical cephalexin NT hydrogel on day 10 compared to plain drug hydrogel • No weight loss and death in NT hydrogel group, normal hair growth and skin appearance • 20% weight loss in plain hydrogel, 20% weight loss and 20–40% suppuration in chitosan-treated and untreated rats	[116]
SLN-based gel	Linezolid	206.3 ± 0.17 ^a^	-24.4 ± 4.67	80.90 ± 0.45	*S. aureus* ^*^	Tape-stripping rat skin infection model	• Bacterial count in LZD SLN-loaded gel treated group was significantly lower compared to negative control group (*P* < 0.0001).	[146]
Cationic NLC	Oxacillin	177.00 ± 9.55 ^b^	18.70 ± 0.82	76.8 ± 7.0	*S. aureus* (ATCC 6538), MRSA (ATCC 33591 and KM1)	BALB/C female mice	• Topical NLC and NLC + oxacillin significantly cleared the skin abscess, with resulting skin texture comparable to healthy skin • NLC and NLC + oxacillin significantly reduced TEWL compared to free oxacillin • 1.0 × 10^4^ reduction of CFU/mL compared with the untreated control • Attenuation of neutrophil accumulation after nanoparticle treatment compared to topical oxacillin	[141]
Polymeric nanoparticle	5-Nitro-2-(3-phenylpropoxy) pyridine (5-NPPP)	281.7 ^c^	+ 8.39	90.03 ± 0.50	*S. aureus* (Sa-1199B)	BALB/C mice	• Weight loss of mice model in infected control group but not observed in treatment group due to clearance of infection • Dual drug-loaded hydrogels showed the events of re-epithelialization while CIP-loaded hydrogels showed the events of infection similar to diseased control • In vivo serial passage assay: Combinatorial treatment (5-NPPP and CIP) significantly reduced CFU/mL of fluoroquinolone-resistant strain	[153]

Abbreviations: Ciprofloxacin, CIP; Colony-forming unit, CFU; Dialdehyde nanocrystalline cellulose, DNC; Fusidic acid, FA; Injured skin, IS; Levocetirizine, LVC; Linezolid, LZD; Liposome, LP; Luteolin, LUT; Methicillin-resistant *Staphylococcus aureus*, MRSA; Minimum bactericidal concentration, MBC; Minimum inhibitory concentration, MIC; Nanoemulsion, NE; Nanoparticle, NP; Nanostructured lipid carrier, NLC; Transfersome, NT; 5-Nitro-2-(3-phenylpropoxy) pyridine, 5-NPPP; Normal skin, NS; Silver nanoparticle, AgNP; Skin colonised by MRSA, SC; Soyaethyl morpholinium ethosulfate, SME; *Staphylococcus aureus*, *S. aureus*; *Staphylococcus epidermidis*, *S. epidermidis*; Terpesome, TP; Transepidermal water loss, TEWL; Zinc oxide nanoparticle, ZnONP; Zone of inhibition, ZOI

^a^ Size measured using dynamic light scattering (DLS)

^b^ Size measured using transmission electron microscopy (TEM)

^c^ Size measured using scanning electron microscopy (SEM)

^*^ Isolate used unspecified

The studies included in this review have primarily investigated the in vivo antibacterial efficacy of optimised topical nanoformulations against *S. aureus* (Fig. 7c). The trend aligns with the updated WHO Bacterial Priority Pathogens List released in May 2024, which classifies *S. aureus* as a high-priority pathogen due to concerns over antimicrobial resistance, while Group A and Group B *Streptococci* are categorised under the medium-priority group [176]. Despite numerous in vitro studies conducted on Gram-negative bacteria, none have been successfully translated into in vivo models at the time of writing. Notably, *P. aeruginosa* is classified as a high-priority pathogen due to its growing multidrug resistance, accentuating the urgent need to overcome treatment challenges. Herein, it is strongly emphasised that future research further pursues the generation of in vivo data involving Gram-negative bacteria to address current gaps and support the development of topical nanoformulations for SSTI management.

In conclusion, this review perceived the direction of research for the development of topical treatments for bacterial SSTIs to be shifting towards topical nanoformulations as a result of satisfactory outcomes and tolerability. Some studies even compared their formulations with the marketed counterparts which was able to generate comparative data that appeared to be more convincing for transition into clinical trials. Recent literature increasingly emphasises investigations into dermal irritation, skin permeation, and anti-biofilm activity of topical nanoformulations. To realise the successful clinical translation of these formulations, it is imperative to resolve the existing ambiguities concerning their safety profiles, therapeutic efficacy, and cutaneous retention characteristics. In acknowledgment of the shift, more studies in future are required to expand the current library of topical nanoformulations against SSTIs in order to impel the clinical progression as the top-priority requisite.

## Data Availability

More information regarding the data presented in this review will be available upon request.

## Abbreviations

CFU: Colony-forming unit
FA: Fusidic acid
FIC: Fractional inhibitory concentration
MRSA: Methicillin-resistant Staphylococcus aureus
MBC: Minimum bactericidal concentration
MIC: Minimum inhibitory concentration
NP: Nanoparticle
P. aeruginosa: Pseudomonas aeruginosa
SSTI: Skin and soft tissue infection
S. aureus: Staphylococcus aureus
ZOI: Zone of inhibition
